# A Critical Review Examining the Characteristics of Modified Concretes with Different Nanomaterials

**DOI:** 10.3390/ma17020409

**Published:** 2024-01-13

**Authors:** Mohammad Mohtasham Moein, Komeil Rahmati, Ashkan Saradar, Jaeyun Moon, Moses Karakouzian

**Affiliations:** 1 Department of Civil Engineering, Allameh Mohaddes Nouri University, Nour 4641859558, Iran; m.mohtasham.moein@gmail.com; 2 Department of Civil Engineering, Somesara Branch, Islamic Azad University, Somesara 4361947496, Iran; komeil_rahmati@msc.guilan.ac.ir; 3 Department of Civil Engineering, University of Guilan, Rasht 419961377, Iran; 4 Department of Mechanical Engineering, University of Nevada, 4505 S Maryland Pkwy, Las Vegas, NV 89154, USA; jaeyun.moon@unlv.edu; 5 Department of Civil and Environmental Engineering and Construction, University of Nevada, Las Vegas, NV 89154, USA

**Keywords:** nanomaterials, nano-CaCO_3_, nano-clay, nano-TiO_2_, nano-SiO_2_, concrete

## Abstract

The movement of the construction industry towards sustainable development has drawn attention to the revision of concrete. In addition to reducing pollution, the use of nano-materials should lead to the provision of higher quality concrete in terms of regulatory items (workability, resistance characteristics, durability characteristics, microstructure). The present study investigates 15 key characteristics of concrete modified with nano-CaCO_3_, nano-clay, nano-TiO_2_, and nano-SiO_2_. The results of the study showed that nanomaterials significantly have a positive effect on the hydration mechanism and the production of more C-S-H gel. The evaluation of resistance characteristics also indicates the promising results of these valuable materials. The durability characteristics of nano-containing concrete showed significant improvement despite high dispersion. Concrete in coastal areas (such as bridges or platforms), concrete exposed to radiation (such as hospitals), concrete exposed to impact load (such as nuclear power plants), and concrete containing recycled aggregate (such as bricks, tiles, ceramics) can be effectively improved by using nanomaterials. It is hoped that the current review paper can provide an effective image and idea for future applied studies by other researchers.

## 1. Introduction

The use of land in the direction of human development can have a significant change on the planet [1,2,3]. These changes can be divided into two groups: (1) compensable and (2) non-compensable. Some of these damages and changes to forest and agricultural areas can be offset, but only if activities are stopped and restoration and replanting measures are taken [4]. On the other hand, the changes that have replaced the physical structure of vegetation are considered by many to be irreversible [5]. In this regard, metal and concrete infrastructures have caused some of the most lasting changes on the planet [4]. 

**Figure 1:**
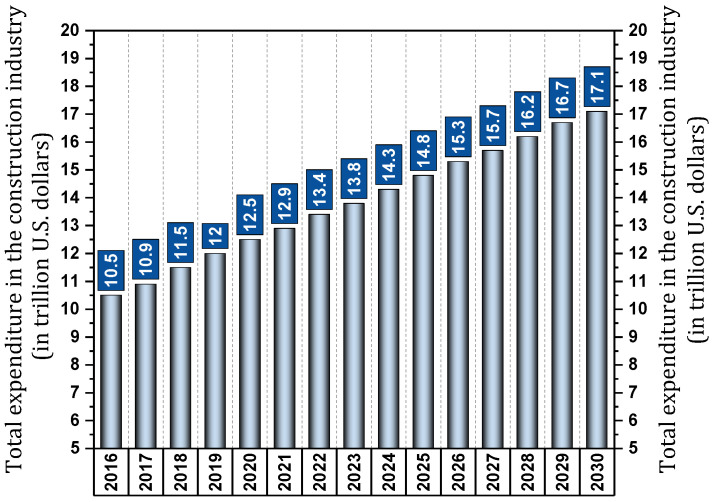
Total construction costs (residential and non-residential) in the world during different years [7].

The world population will reach more than 9972 million people by 2050 [6]. One of the factors that increase construction activity is population. According to Figure 1, which schematically shows the Statista [7] report on total construction costs (residential and non-residential construction costs) in different years, construction costs reached $10.5 trillion in 2016, $10.9 trillion in 2017, and $11.5 trillion in 2018 [7,8]. These costs are projected to reach $14.8 trillion by 2025 [7,8].

Today, there is an increasing interest in reviewing the industry and construction and how to use materials in a way that has minimal damage to the environment [9,10,11]. In line with these revisions, concrete is at the center of attention as a widely used construction material [12,13,14]. In this context, nanotechnology has attracted the attention of the construction industry due to its exceptional potential and properties that it has demonstrated in various industries.

## 2. Research Significance

Population and construction growth are directly proportional to each other. The concrete manufacturing process cannot be considered an environmentally friendly process. Cement alone, which is only one of the constituents of concrete, causes 5–7% of carbon dioxide (CO_2_) emissions on the planet. It seems that the potential hidden in nanomaterials can appear in the role of improving cement defects and accelerating sustainable development. In this regard, this study examines the comprehensive characteristics of concretes (in some cases, mortars) made with different percentages of four types of nano (nano-CaCO_3_, nano-clay, nano-TiO_2_, and nano-SiO_2_). In addition, 15 important items for nano-containing concretes were investigated in different studies. The items under review include: (1) slump, (2) compressive strength, (3) flexural strength, (4) tensile strength, (5) impact strength, (6) water absorption, (7) chloride penetration, (8) carbonation, (9) acid attack, (10) sulfate attack, (11) freeze and thaw, (12) electrical resistivity, (13) elevated temperature, (14) shrinkage, and (15) microstructure. Finally, research gaps and suggestions for future research were presented, which will hopefully be useful.

## 3. Methodology

**Figure 2:**
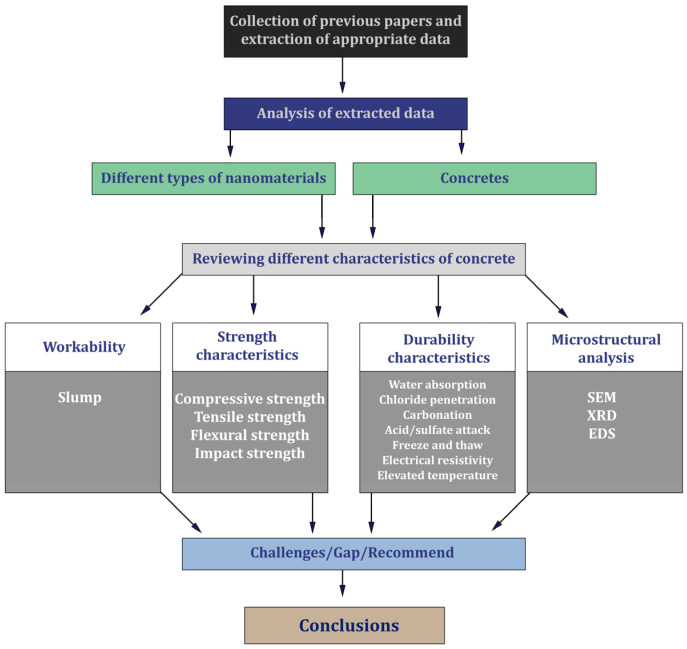
The flowchart diagram describing the methodology utilized in this study.

In recent years, various research has been conducted on the use of nanomaterials in concrete. Considering the importance of sustainable development for the construction industry, the present study investigates four different nanomaterials. In general, four main items (including workability, resistance characteristics, durability characteristics, and microstructure) and 15 sub-items (including slump, compressive strength, tensile strength, flexural strength, impact strength, water absorption, chloride penetration, carbonation, acid attack, sulfate attack, freeze and thaw, electrical resistivity, elevated temperature, shrinkage, and SEM/XRD/EDS) were investigated for each concrete containing these four nanomaterials. Figure 2 shows the flowchart that summarizes further facts regarding the approach of this study.

## 4. Construction Industry: Prospects and Challenges 

**Figure 3:**
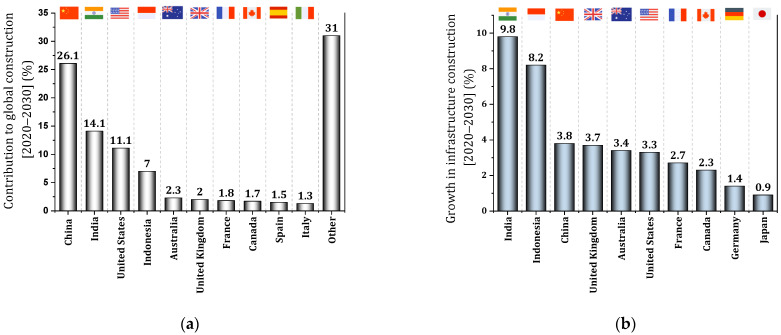
Information about the construction industry from 2020 to 2030 [15]: (**a**) The growth of construction production in different countries; (**b**) The growth rate of infrastructure in different countries.

Figure 3a shows the growth of construction production in different countries between 2020 and 2030 [15]. According to the report of Oxford Economics [15], about 58.3% of the global growth in the construction field from 2020 to 2030 belongs to four countries: China, India, The United States of America (USA), and Indonesia. In this regard, China is at the top of the list with a global growth of 26.1%. India and America are ranked 2^nd^ and 3^rd^, respectively, with global growth of 14.1% and 11.1%. Indonesia then ranks fourth with a global growth of 7%. The combined global growth of Australia, the UK, France, and Canada is equal to the global growth expected in Indonesia. It is predicted that the global production of construction in the period of 2020–2030 will be about 35% more than the period of 2010–2020 [15]. It is expected that among the different sectors in the construction industry until 2030, infrastructure will be the sector that will have the fastest growth [15,16]. Figure 3b shows the growth rate of infrastructure from 2020 to 2030 for different countries [15].

**Figure 4:**
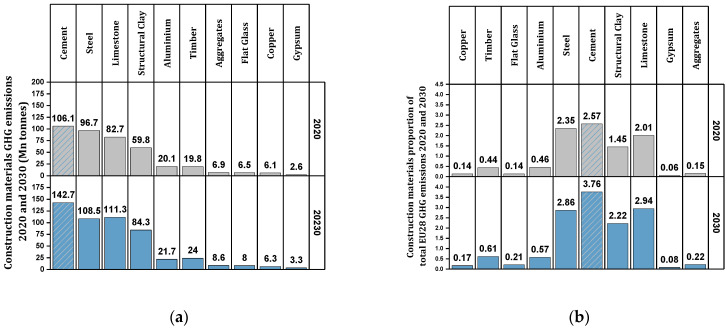
Greenhouse gas (GHG) emissions in the construction industry from 2020 to 2030 [15]: (**a**) The ratio of construction materials to total GHG; (**b**) The amount of GHG emissions by construction materials.

**Figure 5:**
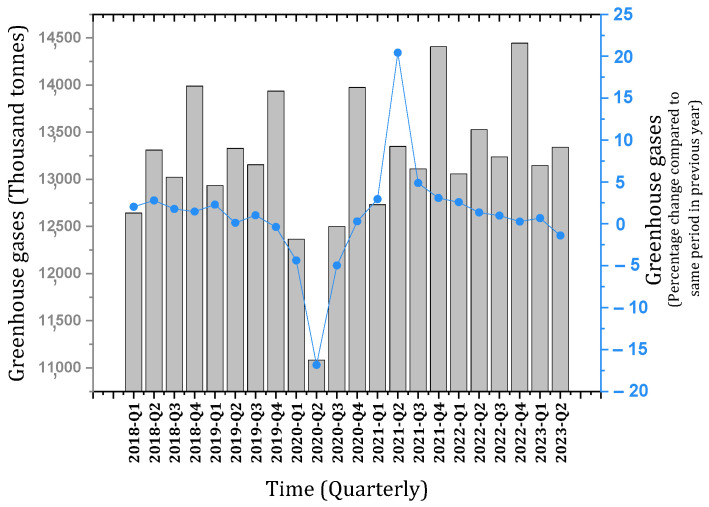
Greenhouse gas (GHG) emissions related to construction in Europe [19].

The program considered by the European Commission in the direction of sustainable development is to transform Europe into a carbon-free continent by 2050 [15,17]. Also, the European Commission intends to reduce about 55% of greenhouse gas emissions by 2030 compared to 1990 [15,18]. Reports indicate that if effective measures are not taken, 10 common building materials in European countries will impose about 518 million tons of greenhouse gases on the planet by 2030 [15]. Figure 4a shows the amount of greenhouse gas emissions by construction materials in Europe in 2020 and the forecast for 2030 [15]. Figure 4b shows the ratio of construction materials to total greenhouse gas emissions in 2020 and the forecast for 2030 [15]. Figure 5 schematically shows the Eurostat report [19] about greenhouse gas emissions of the construction industry in the European continent. This huge wave of construction around the world can bring the planet to the brink of destruction in the not-too-distant future. The measures taken so far have not been effective enough to compensate for the damages caused to the environment.

Concrete is considered one of the oldest building materials in the world [20]. From the past until now, this valuable material has played a significant role in the development of modern society, which includes the construction of road networks, water supply systems, construction of buildings and structures, dams, bridges, and health infrastructure [20,21,22,23]. Concrete is at the top of the list of the most popular artificial materials in the world [24,25,26]. After water, concrete is the most consumed material on the planet [27,28]. Concrete consists of three elements, which include [29,30,31,32] (1) aggregate (such as gravel, sand, and crushed stone), (2) cement (usually Portland cement), and (3) water. When dry cement, aggregate, and water are combined, a fluid mixture is created that has the potential to mold the desired mold [33,34]. The resulting mixture hardens with the help of a chemical process called hydration and, finally, concrete is produced [35]. During the hydration process, crystals play an important role as they bind together and thus bind the components together to obtain dense concrete [34,36].

Humans used concrete-like materials in the past, and numerous examples of the use of cement adhesives have been reported in previous civilizations [4]. Examples of these structures include [4,37] (1) the city of Çatalhöyük which was built 9000 BC, (2) a religious structure in Anatolia which was built 10,000 to 120,000 BC, and (3) a two-layered concrete floor in Galilee that dates back to 7000 BC. Hydraulic cement was first developed by the Greeks (700–600 BC) and later expanded greatly by the Roman Empire [4,38]. Before the Industrial Revolution in Britain, the quality of concrete products was not very favorable and was rarely used [4]. John Smeaton (in 1759) revolutionized the concrete industry when he built a new lighthouse, Smeaton’s Tower, on Eddystone Rock [37,39]. Later, Joseph Aspdin patented Portland cement [4,37]. Most of the concrete production occurred after 1950, and, from 1995 until now, concrete production has experienced rapid growth [4]. 

The concrete production process is not considered an environmentally friendly process. One of the important components of concrete is cement [40]. The production of 1 ton of concrete imposes about 1 ton of CO_2_ on the environment [41]. The Cement Association of Canada (CAC) reported that in 2007, cement production was about 2.7 billion tons [42]. According to reports, in 2009, the cement industry was responsible for 5% of global CO_2_ emissions [43]. The annual use of concrete is between 25 and 30 gigatons [44,45]. Examining the amount of concrete consumption between 2011 and 2013 in China indicates the consumption of 6.6 gigatons of concrete [46,47]. Due to the mass production of concrete, a huge environmental burden is brought to the planet, which includes waste production, greenhouse gas emissions, pollution, etc.

## 5. Nanotechnology

The National Nanotechnology Initiative (NNI) in the United States defines nanotechnology as science, engineering, and technology that is carried out at the nanoscale (1–100 nm) (the upper and lower limits of this range are chosen according to the agreement) [48]. Also, Roco et al. [49] provided another definition that emphasizes that nanotechnology has the potential to manage and reconstruct matter at the atomic and molecular level in the range of 1 to 100 nm and exploit its distinctive properties.

**Figure 6:**
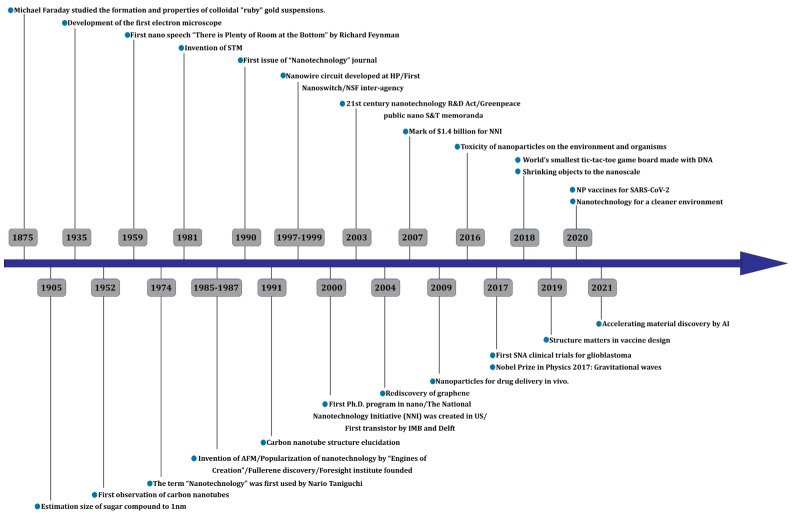
A timeline of nanotechnology development.

Figure 6 shows the timeline of the development of nanotechnology [50,51,52]. The beginning of this path and the idea of nanotechnology was proposed by Richard Feynman in 1959 in a speech called “There’s Plenty of Room at the Bottom” [53,54]. But before 1974, a special name was not considered for this set of debates. In 1974, Norio Taniguchi used the term nanotechnology for the first time [48,55]. About 10 years later, Drexler used the term nanotechnology in his book Engines of Creation: The Coming Era of Nanotechnology, inspired by Richard Feynman’s idea [48,56].

After the emergence of progress due to the use of nanotechnology in the biomedical and electronic industry, the construction industry realized the huge potential hidden in nanotechnology and looked for a way to use these nanomaterials in the construction of more efficient materials [57,58]. The Strategic Development Council from the USA states has identified four separate categories of important and vital research and research areas that are necessary for the advancement of the industry, which include these items [59]: (1) Design and Structural Systems, (2) Constituent Materials, (3) Concrete Production, Delivery, and Placement, and (4) Repair and Rehabilitation. In the constituent materials category, research needs were divided into three groups [59]: (1) new materials, (2) measurement and prediction, and (3) reuse and recycling. Nanomaterials are included in the category of new materials and are among the cases that need more research, especially their use in the concrete industry. 

## 6. Nanomaterials in Construction: Opportunity or Threat?

It should be pointed out that construction activities (such as the construction of structures and elements, repair, renovation, or demolition) can lead to the release of some nanomaterials into the environment. In this regard, the standard methods of destruction of devices to dispose of hazardous substances (such as lead-based paint, asbestos cement, and some persistent residues) should be considered [57,60]. Implementation of these methods usually requires an expert team. For example, sensor devices or coated windows, which are a sub-branch of construction products with nano, should be removed with the utmost caution [57].

After the stage of demolition of the buildings, it is time to transport and unload them at the disposal sites. Waste disposal sites are a suitable place for the environmental release of solid waste nanomaterials. At the disposal site, the waste will undergo a crushing process. Landfarming, landfilling, and incineration can be mentioned among the common ways of environmental emission of nanomaterial solid waste [57,61]. This process can cause aerosolization of nanomaterial waste (that is, the transformation of physical materials into very small and very light particles so that they have the potential to be suspended in the air) [57]. Although it is thought that the nanomaterials used in composites do not pose a significant risk, it seems that using the correct process of using these materials is associated with dangers. There are reports about these pollutions, which include (1) tin in ship hull paint [62], (2) antifouling paint particles [63], and (3) Ni-Cd from batteries [64], bisphenol from food containers f [65], asbestos from tiles [66]. The extent of these pollutions is not large at present, but if the requirements to control these pollutions are not taken into account, it will cause serious problems for the planet in the long term.

**Figure 7:**
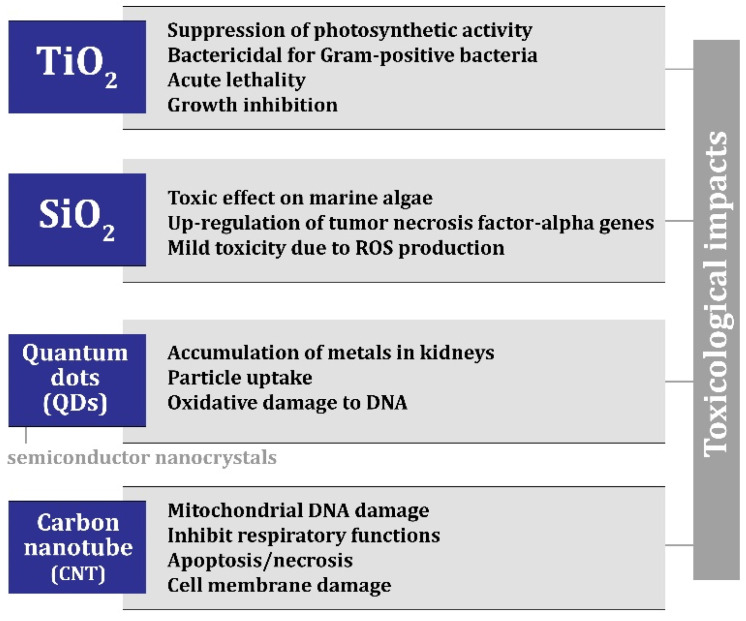
The toxicological effects of nanomaterials.

In the construction industry, according to the studies conducted on nanomaterials, it has been concluded that there is a promising future for moving more toward nanotechnology. However, it should be noted that an incorrect process when applying nanomaterials can turn the opportunity for upgrading and improving building materials into a loss. Nanomaterials that are released from building materials are capable of exposing microorganisms to toxicological risk. Figure 7 shows the toxic risks caused by nanomaterials [57,66,67,68,69,70,71,72,73]. Anastas and Zimmerman [74] suggested principles for the safer use of nanomaterials.

## 7. Nanomaterials in Concrete

### 7.1. Nano-CaCO_3_

The origin of nano-CaCO_3_ can be chalk, marble, or limestone [75,76]. Another way to achieve this type of nano is its artificial production through the combination of Ca and CO_2_ [77]. One of the parameters that make this nanomaterial very popular is its low price compared to other nanomaterials. Apart from the economic discussion, nano-CaCO_3_ can stabilize the formation of ettringite and accelerate the hydration of tricalcium silicate and dicalcium silicate at a young age [77,78]. The paper, paint, food, construction, automotive, and plastic industries are among those that use nano-CaCO_3_ [79,80,81].

**Figure 8:**
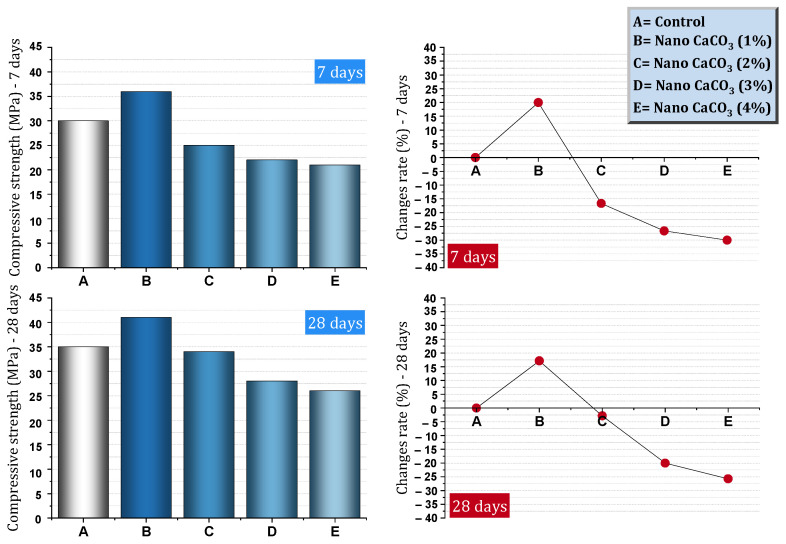
Compressive strength results of mixtures containing nano-CaCO_3_ (7 and 28 days).

Table 1 shows a report of the details and important results of studies conducted on concrete (in some cases cement mortar) containing nano-CaCO_3_. Shaikh et al. [82] reported that the use of 1% nano-CaCO_3_ led to an increase in compressive strength at early ages by 146–148% more than the control mixture (Figure 8). At the age of 90 days, the process of improving the compressive strength by nano-CaCO_3_ continued, so that about 40% of the compressive strength of the mixture was higher than the control mixture. At the ages of 28 and 90 days, the mixture containing 1% nano-CaCO_3_ decreased water sorptivity by 17% and 30%, respectively. They noted that the volume of permeable voids significantly decreased by 1% nano to 46% at the age of 28 days. At the ages of 28 and 90 days, the chloride ion permeability for mixtures containing 1% nano-CaCO_3_ decreased by 20% and 50%, respectively, compared to the control mixture. Due to the use of 1% nano-CaCO_3,_ the chloride diffusion coefficient decreased by about 73%. They pointed out that the presence of nano-CaCO_3_ effectively reduced capillary porosities and refined pores.

Camiletti et al. [83] investigated the characteristics of the early ages of ultra-high-performance concrete (UHPC) containing nano-CaCO_3_. They reported that nano-CaCO_3_ improves flowability and also has a significant effect on the setting and hardening of UHPC at early ages. They mentioned nano-CaCO_3_ as a setting and hardening accelerator. Compressive resistance was reduced by using high doses (15%) and they attributed this issue to the dilution effect. The dose of 5% and 10% was introduced by them as the appropriate dose for the efficiency of concrete properties. Ghabban et al. [84] reported the improvement of the mechanical properties of concrete by nano-CaCO_3_. Also, among the dosages used (0%, 1%, 2%, 3%, 4%) in their study, they introduced a 4% dosage as the optimal dosage for improving mechanical properties. The reduction of flowability and the increase of the heat of hydration was another result that was mentioned. They stated that flowability for nano-CaCO_3_ mixtures is higher than for nano-SiO_2_ mixtures. 

**Figure 9:**
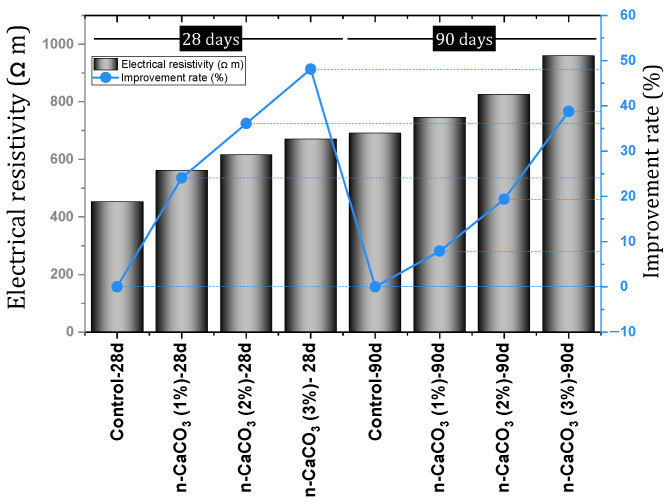
The effect of nano-CaCO_3_ on electrical resistivity (28 and 90 days).

**Figure 10:**
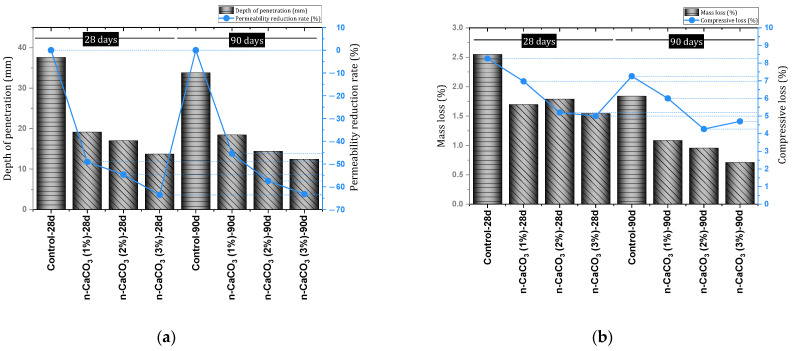
(**a**) The effect of nano-CaCO_3_ on permeability (28 and 90 days). (**b**) Performance of nano-CaCO_3_ against freeze and thaw cycles (28 and 90 days).

Wang et al. [85] investigated the static and dynamic characteristics of concrete containing nano-CaCO_3_ and nano-SiO_2_. They reported that nano-CaCO_3_, in addition to improving the compressive, tensile, and flexural strength of concrete, results in higher strength than mixtures containing nano-SiO_2_. They reported that nano-CaCO_3_ improved the dynamic compressive strength, peak strain, impact toughness, and energy dissipation. Farokhzad and Divandari [86] investigated self-compacting concrete (SCC) containing nano-CaCO_3_. They concluded that the best dosage for improving compressive strength by nano-CaCO_3_ is 3%. They also reported the improvement of tensile strength and indicated that the results of 90 days of age show a more obvious improvement from the effect of nanomaterials (the best dose for tensile strength was 3%). According to Figure 9, the electrical resistivity increases with the addition of nano-CaCO_3_ at the ages of 28 and 90 days. Reducing permeability (Figure 10a) and improving resistance to freeze and thaw cycles (Figure 10b) were other results of this study. They concluded that the best dosage of nano-CaCO_3_ to reduce permeability is 3% and, to improve resistance to freeze and thaw cycle, 2%.

Feng et al. [77] investigated high-strength concrete (HSC) under the influence of nano-CaCO_3_. They reported that nano-CaCO_3_ effectively improved early-age free and restrained shrinkage. The mixture containing 1% nano showed the best performance in reducing the free and restrained shrinkage of concrete. Also, this mixture showed a significant effect in reducing residual stress. The improvement of compressive strength, tensile strength, and elastic modulus was also reported (the best dosage is 1%). They noted that using a dose higher than 1% increased the cracking potential of HSC. Among the other results of this study, we can mention the reduction of relaxed stress and tensile creep due to the use of a 1% dose of nano-CaCO_3_ (but the use of 2% and 3% doses caused an increase). Salih et al. [87] investigated the effect of temperature (25–800 °C) on cement paste containing nano-CaCO_3_. They reported that the use of 1% of nano-CaCO_3_ prevented 76% of cement mass loss at 800 °C. The use of nano-CaCO_3_ also increased the shear stress limit and the yield stress. 

**Figure 11:**
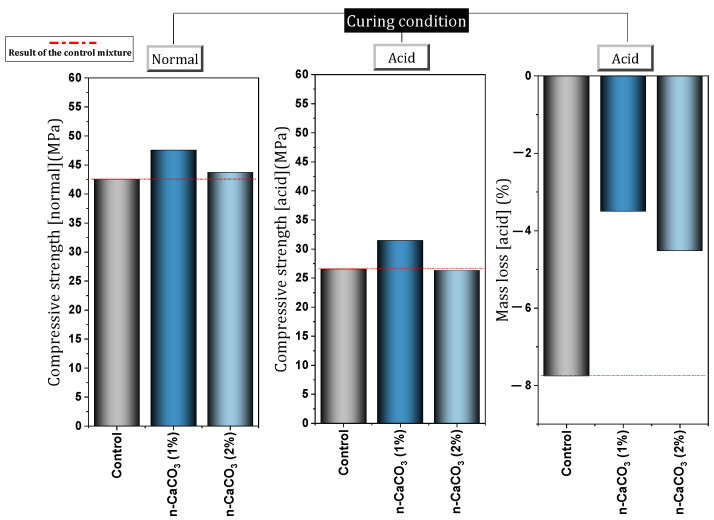
Results of compressive strength and mass loss of mixtures containing nano-CaCO_3_ exposed to acid.

Qiao et al. [88] investigated concrete containing different percentages (0%, 1%, 2%, 3%, 4%, and 5%) of nano-CaCO_3_. They reported that the addition of 1% increases the resistance of concrete against sulfate attack and can improve the useful life of the structure. Bankir et al. [89] investigated the effect of using nano-CaCO_3_ in a mortar containing slag in an acidic environment. They report that the use of a 1% dose of nano-CaCO_3_ improves the compressive strength, flexural strength, and resonance frequency of mortar. Also, the improvement of acid resistance was achieved by using a 1% dose of nano-CaCO_3_. In this regard, the used nanomaterials prevented about 4.2% mass loss. Figure 11 shows the performance of the mixture containing nano-CaCO_3_ exposed to the acidic environment. Li et al. [90] investigated autoclaved concrete containing 0%, 1%, 2%, and 3% of nano-CaCO_3_. They concluded that the use of 3% nano-CaCO_3_ has the best effect on improving the long-term carbonation or chloride resistance of concrete. In this regard, it was reported that the mixture containing 3% nano-CaCO_3_ increased the resistance by 66.8% and 70.8% in the fields of carbonation resistance and chloride resistance, respectively. It was also stated that the mechanism of action of nano-CaCO_3_ in improving the durability of concrete includes (1) reducing porosity and proportion of large pores, (2) purifying micropores and improving their structure, and (3) accelerating the hydration process. For more studies on the nano-CaCO_3_, see Appendix A.

**Table 1:** A summary of studies based on nano-CaCO_3_.

N.O.	n-CaCO_3_ (%)	CS	TS	FS	Other Reviews	Results	Ref.
1	0, 1, 2, 3, 4	Y	N	N	Slump, Water sorptivity, Volume of permeable voids, RCPT, Chloride diffusion, SEM, XRD, MIP, DTA/TG	Improving compressive strength (the best dosage is 1%): Early ages = about 148–146% improvement, Age 90 days = about 40% recovery, Reduction of water absorption (the best dosage is 1%): 28 days = 17% reduction, 90 days = 30% reduction, Reduction of permeable voids (by 46%), Reducing the chloride ion permeability (the best dosage is 1%): 28 days = 20%, 90 days = 50%, Reducing the chloride diffusion coefficient (by 73%), Effectively reduces capillary porosities and refines pores	[82]
2	0, 5, 10, 15	Y	N	N	Setting time, Heat of hydration	Improve flowability, Significant effect on the setting and hardening	[83]
3	0, 1, 2, 3, 4	Y	Y	Y	Slump, Water absorption	Improving compressive strength (best dose = 4%), Improving tensile strength (best dose = 4%), Improvement of flexural strength (best dose = 4%), The reduction of flowability and the increase of the heat of hydration	[84]
4	0, 2	Y	Y	Y	Impact test (by SHPB), SEM, MIP	Improving compressive, tensile, and flexural strength, Improving the dynamic compressive strength, peak strain, impact toughness, energy dissipation	[85]
5	0, 1, 2, 3	Y	Y	N	Slump, J-ring, V-funnel, L-box, Electrical resistivity, Freeze and thaw cycle, Permeability, UPV, SEM, XRD	Improving compressive strength (best dose = 3%), Improvement of tensile strength (best dose = 3%), Decreased permeability (best dose = 3%), Improving resistance to freeze and thaw cycle (best dose = 2%)	[86]
6	0, 1, 2, 3	Y	Y	N	Modulus of elasticity, Free Shrinkage, Restrained Ring Test	Improving compressive and tensile strength (best dose = 1%), Improving modulus of elasticity (best dose = 1%), Reduction of free and restrained shrinkage (best dose = 1%), Reducing residual stress, Reduction of relaxed stress and tensile creep (dose 1%)	[77]
7	0, 1	Y	Y	N	Rheological, Raman, FTIR, SEM, XRD, DTA/TG	Preventing weight loss of cement at 800 °C, Increased the shear stress limit and the yield stress, Improved compressive strength	[87]
8	0, 1, 2, 3	N	N	N	Carbonation resistance, Chloride resistance, TG/DTG, SEM, XRD	Improved resistance to carbonation, Improved resistance to chloride, Improved the microstructure of concrete	[90]

CS = Compressive Strength, TS = Tensile Strength, FS = Flexural Strength.

### 7.2. Nano-Clay

Nano-clay can be referred to as nanoparticles with layered mineral silicate structures [91,92]. The use and influence of clay in the construction of human societies has a very long history. The size of clay particles (x) is different in different sciences; for example, geologists and soil scientists consider the size of clay particles to be less than 2 μm (x < 2), sedimentologists consider the size of clay particles to be 4 μm (x = 4), and colloid chemists consider the size of clay particles to be 1 μm (x = 1) [93]. Nano-clay can play the role of an excellent filler for cement, and this is because the average size of nano-clay particles is about 1000 times smaller than cement [94]. Investigations show that nano-clay is able to produce the needle action system due to its particle shape (long, thin, flaky) and favorable pozzolanic activity (alumina 35.3–41.8% and silica 44.98–47.8%) [94,95]. Nano-clays usually include these materials [96,97,98,99,100]: (1) nano-montmorillonite, (2) nano-kaolin, (3) nano-halloysite, and (4) calcined nano-clay (combination of nano-clay and nano-metakaolin).

Table 2 shows a report of the details and important results of studies conducted on concrete (in some cases cement mortar) containing nano-clay. Wang [91] investigated concrete containing different percentages (0%, 0.1%, 0.3%, and 0.5%) of nano-clay. This study showed that concrete containing nano-clay shows more resistance when the temperature does not exceed 300 °C. The temperature in the range of 440 °C to 450 °C led to a significant decrease in the compressive strength of concrete containing nano-clay. Reaching the temperatures of 800 °C and 1000 °C showed that the strength of concrete containing nano-clay is not as good as 30% and 10% of the initial strength. The thermal conductivity coefficient decreases with increasing temperature, but the use of 0.3% and 0.5% nano-clay led to an increase in thermal conductivity coefficients. Hamed et al. [95] investigated the de-agglomeration of nano-clay in concrete using two dispersion techniques (added as-received, added after being dispersed in water by using a bath sonicator). They reported that for both techniques (as-received and sonicated) the optimal replacement value for increasing the mechanical properties is 7.5%. Compressive, tensile, flexural, and bond strengths for sonicated mixtures are about 1.42–3.47 times of as-received technique mixtures. Also, they reported that nano-clay results in improving concrete structure (pozzolanic effect and filling pores). XRD and AFM analysis showed that the nanomaterials of the Sonicated technique have higher reactivity than the as-received technique.

**Figure 12:**
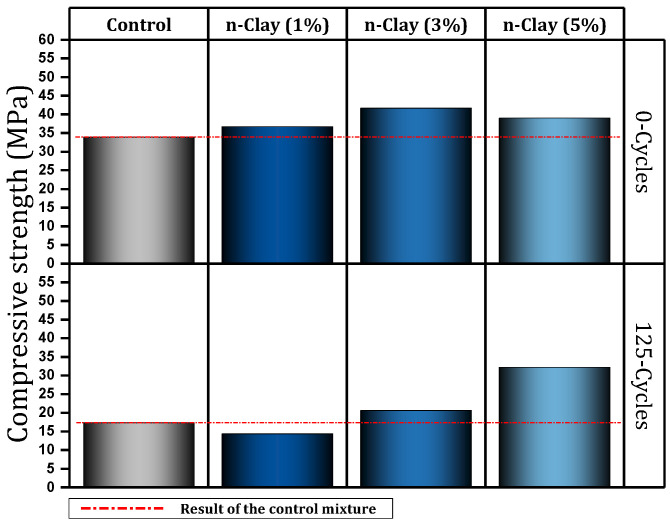
Compressive strength of mixtures containing nano-clay subjected to freeze–thaw cycles (0 and 125 cycles).

**Figure 13:**
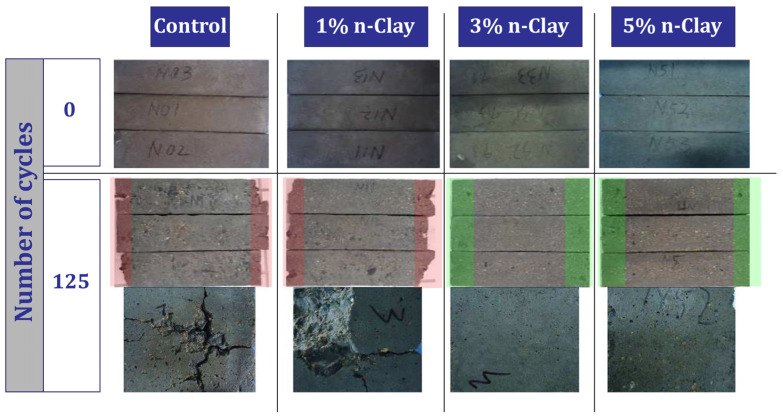
Performance of different mixtures containing nano-clay in 0 and 125 cycles of freeze–thaw [101].

Fan et al. [101,102] investigated freeze–thaw cycles and the bond behavior of concrete containing different percentages (0%, 1%, 3%, and 5%) of nano-clay. After 125 freeze–thaw cycles, the samples were damaged, but the samples containing 3% and 5% nano-clay were in good condition. For example, the mixture containing 5% nano-clay showed about 34% better compressive strength than the control mixture (Figure 12). They concluded rebar corrosion reduction of 53.1% for 5% nano-clay mixtures. Figure 13 shows that after 125 cycles, the control mixture or the mixture containing 1% nano-clay suffered fractures at the edges (red areas). This is despite the fact that in mixtures containing 3% or 5%, only the pores increased and the presence of more nano-clay shows greater resistance to freeze–thaw cycles. 

**Figure 14:**
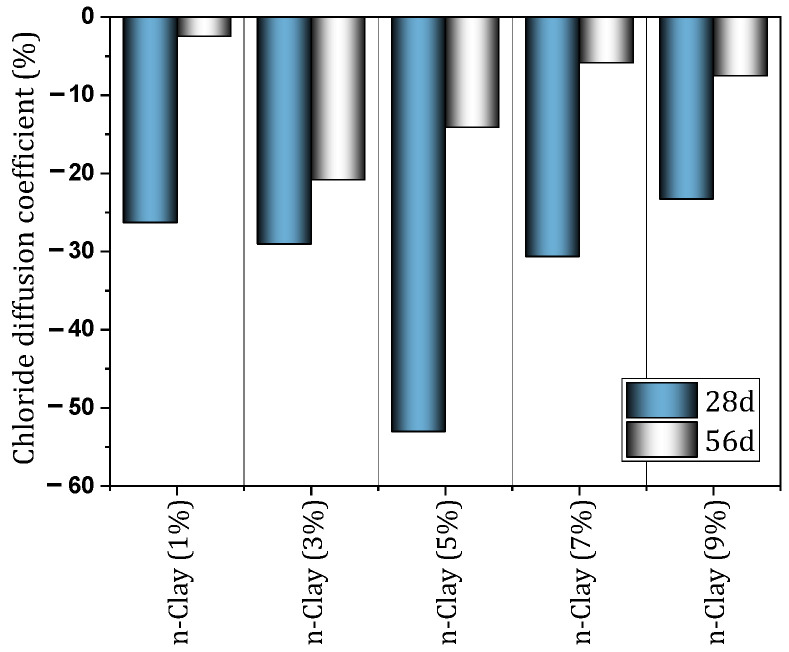
The reduction of the chloride diffusion coefficient for mixtures containing nano-clay.

**Figure 15:**
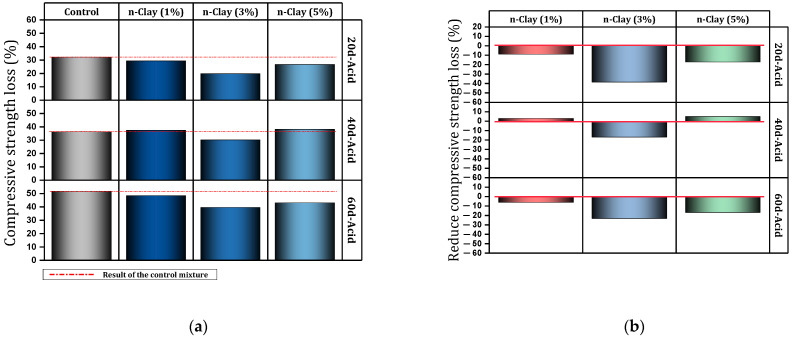
Results of mixtures containing nano-clay exposed to acid: (**a**) compressive strength loss; (**b**) rate of compressive strength loss.

In another study, Fan et al. [103] examined cement-based materials containing different percentages (0%, 1%, 3%, 5%, 7%, and 9%) of nano-clay. They reported that the use of nano-clay reduces flow ability and increases water demand of normal consistency. Also, the use of nano-clay caused a slight reduction in the initial and final setting time. The use of nano-clay caused the resistance to chloride penetration to increase significantly. In this regard, for mixtures containing 1%, 3%, 5%, 7% and 9% of nano-clay, the chloride diffusion coefficient decreased by 27%, 29%, 53%, 31%, and 23%, respectively. Figure 14 shows the reduction of the chloride diffusion coefficient by different percentages of nano-clay. Also, Fan et al. [104] evaluated cement mortar containing 0%, 1%, 3%, and 5% of nano-clay exposed to acid. They cured the cured samples in water for 28 days and then exposed them to acid for 20, 40, and 60 days. Figure 15 shows the performance of the mixture containing nano-clay exposed to an acidic environment. They indicated that the addition of nano-clay prevented a 17% drop in compressive strength for acid-exposed mixes (60 days). They attributed the improvement of mortar resistance to acid attack as a result of the filling and pozzolanic effect of nano-clay particles. The 3% dose recorded the best performance against acid attack compared to other doses.

Liu et al. [105] investigated cement mortar containing nano (nano-attapulgite-clay and nano-metakaolin) in terms of shrinkage cracking morphology. They reported a significant reduction in the number of cracks, crack width, crack length, average cracking area, and unit cracking area of each crack with the addition of nano-clay. In this regard, they reported that in the field of anti-cracking effect, nano-clay showed a better performance than nano-metakaolin. Also, Polat et al. [106] also mentioned the reduction of autogenous shrinkage for cement mortar by 3% nano-clay. Hosseini et al. [107] investigated SCC containing 0%, 0.25%, 0.5%, 0.75%, and 1% of nano-clay. They reported that improvements in compressive and tensile strength were achieved due to the use of nano-clay (the best dosage for compressive strength was 0.5% and for tensile strength was 0.75%). Increasing the content of nano-clay at different ages of 7, 28, and 56 days caused an increase in electrical resistivity (the best dosage is 1%). Adding 0.25%, 0.5%, 0.75%, and 1% nano-clay to SCC decreased the water penetration depth by 17%, 27%, 39%, and 43%, respectively. Also, they mentioned the three main functions of nano-clay in concrete structures: (1) pozzolanic activity, (2) micro-filling, and (3) micro-reinforcing effects. Diab et al. [108] investigated high-strength concrete (HSC) and high-performance concrete (HPC) containing nano (calcined nano-clay and nano-SiO_2_). The addition of nanomaterials improved the resistance of HSC and HPC against sulfate and acid attacks. They reported that after exposure of samples containing nanoparticles to magnesium sulfate (10%) for 360 days, mixtures containing nano-SiO_2_ resulted in a reduction of 18.6% compressive strength loss. However, the mixtures containing 9% calcined nano-clay reduced the compressive strength loss by 41.4%. Shafabakhsh et al. [109] reported that among the doses of 0%, 1%, 2%, and 3% of nano-clay, the best result was obtained for the mixture containing 1% nano. In this regard, compressive strength (28 days), tensile strength (14 days), and flexural strength (28 days) increased by 35%, 34%, and 31%, respectively. Also, water penetration and water absorption decreased by 35% and 54%, respectively. For more studies on the nano-clay, see Appendix A.

**Table 2:** A summary of studies based on nano-clay.

N.O.	n-Clay (%)	CS	TS	FS	Other Reviews	Results	Refs.
1	0, 0.1, 0.3, 0.5	Y	N	N	Thermal conductivity coefficients	Improve compressive strength, Improvement of thermal conductivity coefficients, The best dosage: 0.3% and 0.5%	[91]
2	0, 5, 7.5, 10	Y	Y	Y	Slipping bond strength, Split bond strength, SEM, XRD, AFM	The best dosage for optimal mechanical properties = 7.5%, Compressive, tensile, flexural, and bond strengths: Sonicated technique mixtures about 1.42–3.47 times as-received technique, Improvement of concrete microstructure, Better reactivity of nanomaterials in the Sonicated technique compared to the as-received technique	[95]
3	0, 1, 3, 5	Y	N	N	Freezing-thaw cycle, Accelerated corrosion, Pull-out	Maintaining the good condition of concrete after 125 freeze–thaw cycles (sample containing 3% and 5% nano), Improving bond behavior (preventing rebar corrosion), Improve compressive strength	[101,102]
4	0, 1, 3, 5, 7, 9	Y	N	Y	Workability (Flow ability, water requirement, Setting time, Soundness), Chloride diffusivity, MIP, SEM, XRD, RCM	Decrease Flow ability, Increase water demand of normal consistency, Slight reduction of initial and final setting time, Improving the compressive and flexural strength of mortar, Significant increase in chloride penetration resistance	[103]
5	0, 1, 3, 5	Y	N	N	Simulated acid rain environment, TG/DS, BSEM, XRD	Improving the microstructure (increasing C-S-H and decreasing CH), Increasing resistance to acid attack (best dose = 3%)	[104]
6	0, 3	Y	N	Y	Elastic modulus, Shrinkage	Significant improvement in Shrinkage Cracking, Improving compressive strength, flexural strength, and elastic modulus	[105]
7	0, 0.25, 0.5, 0.75, 1	Y	Y	N	Slump, V-funnel, L-box, Electrical resistivity, Water penetration, SEM, XRD	Reducing slump flow and increasing flow time, Improving compressive strength (best dose = 0.5%), Improvement of tensile strength (best dose = 0.75%), Increase in electrical resistivity (best dose = 1%), Microstructure improvement	[107]

CS = Compressive Strength, TS = Tensile Strength, FS = Flexural Strength.

### 7.3. Nano-Titanium Dioxide (Nano-TiO_2_)

Nano-sized titanium dioxide or nano-TiO_2_ is considered a semiconductor [110,111]. When this nano is subjected to ultraviolet (UV) radiation in the presence of gas or liquid, it shows a behavior similar to a photocatalyst [111]. Therefore, in many studies, nano-TiO_2_ is referred to as a semiconductor photocatalyst. The photo-catalysis property of this type of nano was proposed for the first time by Fujishima and Honda [112,113]. There is a wide range of applications for nano-TiO_2_ (such as use in glass, paint, cement, cosmetics, ceramics, and tiles, etc.) [111]. Nano-TiO_2_ provides a favorable ability to remove pollutants such as CO, NOx, VOCs, chlorophenols, and aldehydes [114,115,116]. For this reason, the use of nano-TiO_2_ in the construction industry (such as tunnels, pavements, buildings, and hospitals) has become very popular [117]. In general, nano-TiO_2_ has three crystalline phases [118]: (1) anatase, (2) rutile, and (3) brookite. Among the three mentioned crystals, the most photocatalytic activity and stability belongs to anatase. It should be noted that nano-TiO_2_ in the form of anatase crystals is the most produced [118].

**Figure 16:**
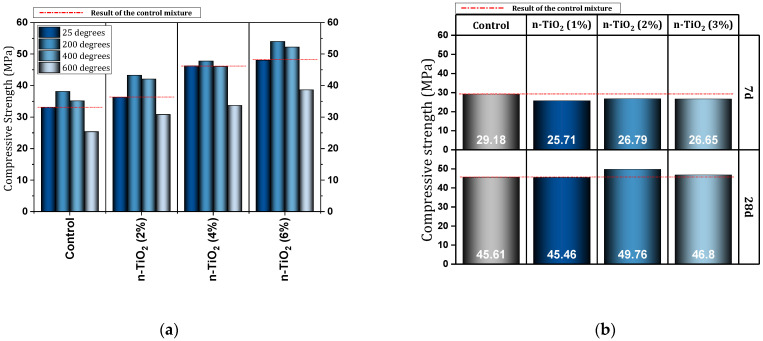
(**a**) Compressive strength of mixtures containing nano-TiO_2_ exposed to temperatures of 25 °C, 200 °C, 400 °C, and 600 °C. (**b**) Compressive strength of mixtures containing nano-TiO_2_ at ages 7 and 28 days.

Table 3 shows a report of the details and important results of studies conducted on concrete (in some cases cement mortar) containing nano-TiO_2_. Nikbin et al. [119] investigated heavy-weight concrete (HC) containing different percentages (0%, 2%, 4%, 6%, and 8%) of nano-TiO_2_. They reported an improvement in the compressive strength of HC due to the use of nano-TiO_2_ (best performance for 6% nano mixture). Mixtures containing 6% and 8% nano-TiO_2_ in the UPV test were in the “excellent” category and the remaining percentages were in the “good” category. In addition, the good performance of nano-TiO_2_ in reducing inner pores was noted. The mixture containing 6% nano-TiO_2_ increased the impact resistance by 35%. The use of nano-TiO_2_ increased the protective effects against gamma rays. In another study, Nikbin et al. [120] investigated the effect of high temperature on the mechanical properties and gamma-ray shielding properties of concrete containing nano-TiO_2_. At a temperature of 200 °C, the mixture containing 2%, 4%, and 6% of nano-TiO_2_ had 13.5%, 25.4%, and 41.7% higher compressive strength than the control mixture, respectively (Figure 16a). At a temperature of 400 °C, the mixture containing 2%, 4%, and 6% of nano-TiO_2_ had 19.8%, 30.99%, and 48.65% higher compressive strength than the control mixture, respectively (Figure 16a). At a temperature of 600 °C, the mixture containing 2%, 4%, and 6% of nano-TiO_2_ had 21.45%, 32.80%, and 52.48% higher compressive strength than the control mixture (Figure 16a), respectively. Also, the lowest amount of compressive strength loss (at 600 °C) was obtained for the mixture containing 2% nano (15.2%). They reported that all the samples after exposure to 600 °C recorded UPV values exceeding 3000 m/s, which places these mixtures in the “doubtful” category.

Joshaghani et al. [121] reported that the use of nano-TiO_2_ in the amount of 3% in SCC improves the workability properties (according to the L-box and V-Funnel results). This is while the use of 5% of this type of nano reduced the workability. They reported the improvement of SCC compressive strength due to the favorable effect of nano-TiO_2_ on the homogeneous formation of C-S-H. The reduction of water penetration depth by nano-TiO_2_ was another result that was mentioned. Reducing the penetration of chloride ions was another achievement of nano-TiO_2_ for SCC. Ren et al. [122] reported that the addition of 3% of nano-TiO_2_ leads to an increase in compressive strength (28 days) by 9%. They reported that the compressive strength of concrete at early ages does not show favorable results with the presence of nano-TiO_2_. Figure 16b shows the compressive strength results at the ages of 7 and 28 days for different mixtures. They also mentioned the filling of pores and the improvement of the paste-aggregate interface by nanomaterials.

Melo and Trichês [123] investigated the concrete containing the separate composition of three types of nano-TiO_2_, including anatase-I (10–30 nm), anatase-II (50–80 nm), and rutile (10  ×  40 nm). Adding 10% of anatase II led to an increase in compressive strength (28 days) by 17.3%. Also, the use of 10% rutile increased the 28-day compressive strength by 10.5%. They reported that the results of the calorimetry test indicate that 10% anatase-II (or 10% rutile) occurs at a larger peak for the maximum heat released and also the accumulated heat of hydration. The control mixture as well as the mixture containing 10% anatase-I showed the highest porosity in terms of SEM. In this regard, the mixture containing anatase-I showed the weakest mechanical characteristics compared to the other two types of nanomaterials. They pointed out that the addition of nano-TiO_2_ reduces the rigidity of concrete and among the types of nanomaterials used, anatase-II or rutile had the best performance. Ying et al. [124] investigated the pore structure and chloride diffusivity of concrete containing recycled aggregates (RA). They reported that due to the nanoparticles used (nano-TiO_2_ and nano-SiO_2_ separately), the refined extent increases first and decreases with the increase of nano content. The use of nanomaterials increased the resistance of concrete against the resistance to chloride diffusion of recycled aggregates concrete (RAC) (the best dosage = 2%). They found the performance of nano-TiO_2_ slightly better compared to nano-SiO_2_ in the field of compressive strength and resistance to chloride penetration. In general, this study recommended the use of nanomaterials to modify the structure of concrete containing waste aggregates in the durability field.

**Figure 17:**
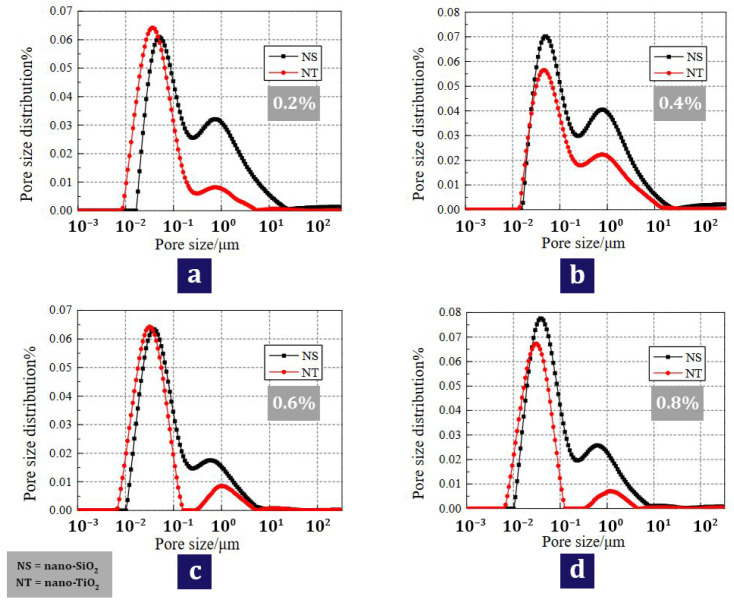
Pore fluid size distribution for mixtures containing nano-SiO_2_ and nano-TiO_2_ under 50 freezing and thawing cycles: (**a**) 0.2%; (**b**) 0.4%; (**c**) 0.6%; (**d**) 0.8% [126].

**Figure 18:**
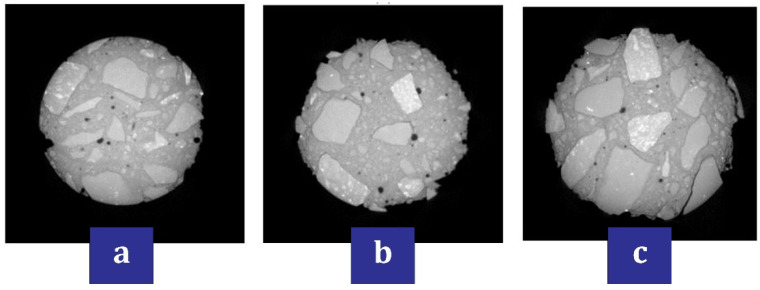
Appearance (CT scanning) of the mixture containing 0.6% nano-TiO_2_: (**a**) 25 cycles, (**b**) 50 cycles, and (**c**) 75 cycles [126].

**Figure 19:**
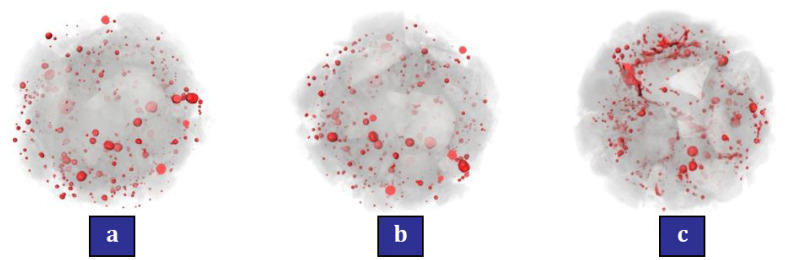
Pore distribution in the mixture containing 0.6% nano-TiO_2_: (**a**) 25 cycles, (**b**) 50 cycles, and (**c**) 75 cycles [126].

Li et al. [125] reported that the use of nano-TiO_2_ can positively increase the scouring abrasion resistance and decrease the chloride ion diffusion coefficient. They recommended that concrete containing nano-TiO_2_ can be a suitable option for marine structures in protecting reinforcing bars against corrosion. Liu et al. [126] investigated the durability and deterioration of concrete containing nano (nano-TiO_2_ and nano-SiO_2_) under freezing and thawing cycles (0, 25, 50, and 75 cycles). According to Figure 17, which shows the pore fluid size distribution of nanomaterials, they pointed out that up to cycle 50, the value of the second peak for the mixtures containing nano-TiO_2_ is smaller than the mixtures containing nano-SiO_2_. This shows that the addition of nano-TiO_2_ has fewer cracks and pores, and as a result, it brings better resistance in terms of freezing and thawing to concrete. Figure 18 shows the CT scanning for the mixture containing 0.6% nano-TiO_2_ in cycles 25, 50, and 75. They pointed out that after 25 cycles, no serious damage is caused to the mixture containing nanomaterials and the only thing that happens is partial exfoliation of the cement paste at the borders of the concrete sample (Figure 18a). In the 50th cycle, the external coarse aggregates start to fall and falter, and after that, the boundaries of the concrete sample become more irregular (Figure 18b). In the 75th cycle, the damages caused by different cycles penetrate into the heart of the concrete and as a result, the peeling of the external cement paste and coarse aggregate becomes more serious (Figure 18c). In Figure 19, the simulation of the occurrence of holes in different cycles can be seen in the mixture containing 0.6% nano-TiO_2_. In cycle 25, pores are mostly irregular spheres (Figure 19a). In cycle 50, pores and cracks expand (Figure 19b). In cycle 75, the pores become more united and surround the coarse aggregates creating gaps in the interface of the cement mortar and coarse aggregate (Figure 19c). They mentioned that nano-TiO_2_ had a better performance than nano-SiO_2_ in preventing and inhibiting pore and crack expansion.

**Figure 20:**
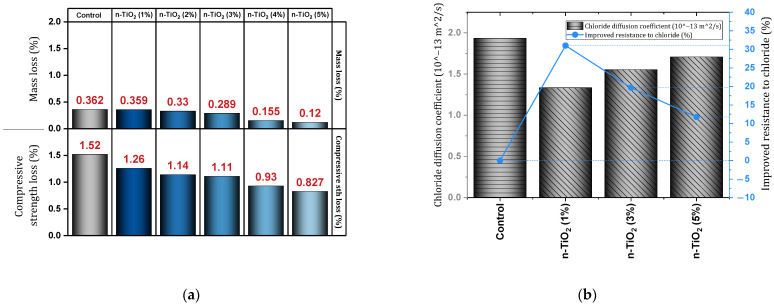
(**a**) Compressive strength and mass loss of mixtures containing nano-TiO_2_ exposed to chloride. (**b**) The reduction of the chloride diffusion coefficient for mixtures containing nano-TiO_2_.

Sastry et al. [127] evaluated the effect of using nano-TiO_2_ (doses of 0%, 1%, 2%, 3%, 4%, and 5%) on geopolymer concrete. Increasing the content of nano-TiO_2_ led to an increase in compressive, tensile, and flexural strength. The use of 5% nano-TiO_2_ improved the compressive, tensile, and flexural strength by 52.3%, 22.2%, and 32.6%, respectively (at the age of 28 days). Also, water absorption and volume of permeable pore space decreased by 15.34% and 19.09%, respectively (5% of nano-TiO_2_). Examining samples exposed to sulfate attack showed that adding 5% of nano-TiO_2_ prevented mass loss and compressive strength loss by 3.293% and 1.36% (Figure 20a), respectively. In addition, examination of the samples exposed to chloride attack showed that the addition of 5% of nano-TiO_2_ prevented mass loss and compressive strength loss by 0.242% and 0.639% (Figure 20a), respectively.

Zhang et al. [128] reported that the pore structure was refined due to the presence of nanomaterials, and the pore structure in the mixtures containing nano-TiO_2_ was finer than the mixtures containing nano-SiO_2_. They pointed out that the addition of nanomaterials resulted in increased resistance to chloride penetration and recommended that the use of a lower content of nanomaterials resulted in better resistance. Figure 20b shows the reduction of the chloride diffusion coefficient by different percentages of nano-TiO_2_. The better performance of nano-TiO_2_ compared to nano-SiO_2_ in dealing with chloride penetration was also revealed. For example, for the mixture containing nano-TiO_2_ (dosage 1%) and the mixture containing nano-SiO_2_ (dosage 1%), the improvement of resistance against chloride penetration was recorded as 31% and 18.04%, respectively. For compressive and flexural strength, the best result was obtained using 1% nano-TiO_2_ (10.28% improvement for flexural strength and 18.03% improvement for compressive strength). Salemi et al. [129] reported the improvement of compressive strength at the ages of 7, 28, and 120 days for the mixture containing 2% of nano-TiO_2_ by 12%, 22.71%, and 27%, respectively. Also, the use of nano-TiO_2_ reduced water absorption by almost 22%. To investigate Frost resistance, four items were evaluated, which include these items (after 300 freezing and thawing cycles): (1) compressive strength loss, (2) change in length, (3) loss of mass, and (4) increase in water absorption. The mixture containing 2% nano-TiO_2_ caused an 88.5% decrease in compressive strength loss, a 26.4% decrease in length loss, and a 78.65% decrease in mass loss as well as preventing an increase in water absorption.

**Figure 21:**
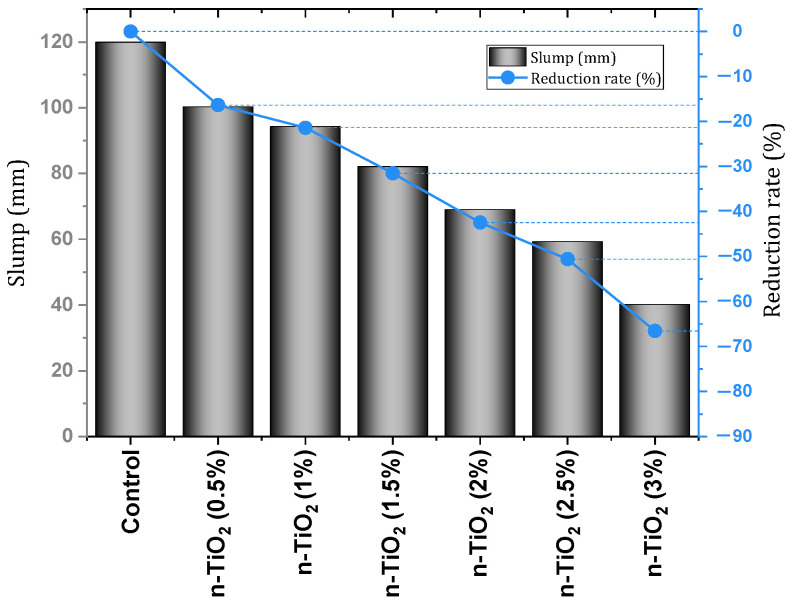
Slump results of mixtures containing nano-TiO_2_.

Rawat et al. [130] investigated the physical, mechanical, and durability properties of concrete containing nano-TiO_2_ (dosage 0% to 3%). In the field of mechanical characteristics, the best result was obtained for the dosage of 1.5%. In this regard, the compressive strength of the mixture containing 1.5% of nano-TiO_2_ improved by 18.67%, 6.45%, 10.5%, and 7.88% at the ages of 7, 28, 56, and 90 days, respectively. The tensile strength of the mixture containing 1.5% of nano-TiO_2_ improved by 19.65%, 16.46%, 13.91%, and 15.25% at the ages of 7, 28, 56, and 90 days, respectively. The flexural strength of the mixture containing 1.5% of nano-TiO_2_ improved by 7.86%, 10.47%, 7.55%, and 5.22% at the ages of 7, 28, 56, and 90 days, respectively. They mentioned that the use of nano-TiO_2_ up to 1.5% dose has the potential to accelerate the formation of C-S-H gel due to the increase in crystalline Ca(OH)_2_ concentration and thus increase tensile and flexural strength at early ages. Meanwhile, the use of doses higher than 1.5% of nano-TiO_2_ leads to a decrease in resistance at early ages due to the reduction of crystalline Ca(OH)_2_ content (necessary for the formation of C-S-H gel). Also, water absorption and apparent porosity decreased significantly, which is because nano-TiO_2_ acts as nanofillers and reduces permeability. The investigation of the ultrasonic pulse velocity (UPV) test indicates that by adding nano-TiO_2_ to concrete, the pores are reduced, which indicates the increase of the density and the improvement of the internal pore structure. It was pointed out that nano-TiO_2_ in concrete produces a more packed microstructure and increases the volume of the paste, which ultimately reduces chloride penetration. According to Figure 21, increasing the content of nano-TiO_2_ decreased the amount of slump.

**Figure 22:**
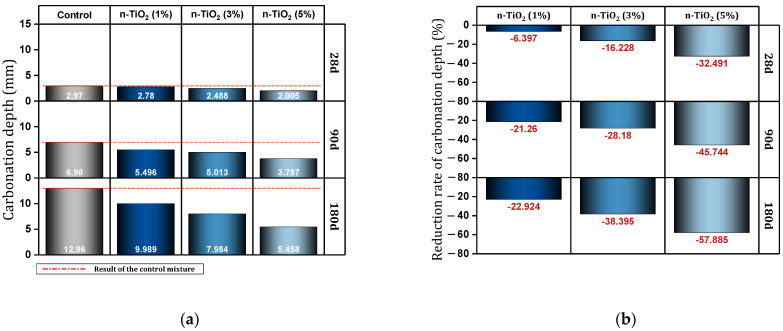
The performance of mixtures containing nano-TiO_2_ against carbonation: (**a**) The carbonation depth, (**b**) The reduction rate of the carbonation depth.

Xu et al. [131] reported that adding 3% of nano-TiO_2_ to concrete improved the resistance of concrete against sulfate attack. In this regard, nano-TiO_2_ decreased compressive loss by 3.87% and mass loss by 2.381%. Duan et al. [132] investigated the effect of nano-TiO_2_ on the characteristics of geopolymer paste (containing fly ash). Figure 22 shows the carbonation depth results of different mixtures aged 28, 90, and 180 days. At the age of 28 days, compared to the control mixture, the mixtures containing 1%, 3%, and 5% of nano-TiO_2_ decreased carbonation depth by 6.40%, 16.22%, and 32.49%, respectively. At the age of 90 days, the mixtures containing 1%, 3%, and 5% of nano-TiO_2_ decrease carbonation depth by 21.26%, 28.18%, and 45.74%, respectively. At the age of 180 days, the mixtures containing 1%, 3%, and 5% of nano-TiO_2_ decrease carbonation depth by 22.92%, 38.39%, and 57.88%, respectively. At the age of 28 days, compared to the control mixture, the mixtures containing 1%, 3%, and 5% of nano-TiO_2_ decreased drying shrinkage by 11.03%, 36.03%, and 48.70%, respectively.

**Figure 23:**
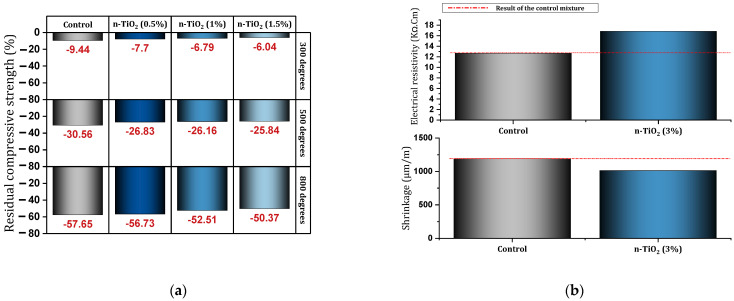
(**a**) Residual compressive strength of mixtures containing nano-TiO_2_ under the influence of temperatures of 300 °C, 500 °C, and 800 °C. (**b**) Result of shrinkage and electrical resistivity of mixtures containing nano-TiO_2_.

Daniyal et al. [133] investigated the fresh, hardened, and microstructural properties of cement composites containing nano-TiO_2_. They reported that SEM micrograph examination showed that cement composites containing nano-TiO_2_ produced a large amount of C-S-H gel and a small number of pores and needle-like crystals compared to the control mixture. The addition of nano-TiO_2_ increased the particle-packing density in cement composites. For mixtures containing nano-TiO_2_, the corrosion rate was much lower than the control mixture. The use of 5% nano-TiO_2_ was introduced as the optimal dose to deal with corrosion, but even the 3% dose shows a very high inhibition efficiency. The 360-day compressive strength of the mixture containing 5% nano-TiO_2_, which was exposed to an acidic environment, was recorded as 25.80% higher than the compressive strength of the control mixture. Fattah et al. [134] also reported in their study on cement composites containing 2%, 6%, and 10% of nano-TiO_2_ that the presence of this nano can make the cement composite resistant to acid attack. Gopalakrishnan et al. [135] investigated cement mortar containing different percentages of nano-TiO_2_ under industrial wastewater treatment. Acceleration of the hydration process, improvement of the pore structure, and reduction of mortar porosity were reported due to the use of nano-TiO_2_. Also, an increase in the electrical resistance of cement mortar was recorded at the ages of 3, 7, and 28 days. In this regard, a 33.70%, 28.50%, and 42.50% increase in electrical resistance were recorded for the mixture containing 2% nano at the ages of 7, 28, and 90 days, respectively. In addition, 93.87%, 77.19%, and 105.61% increases in electrical resistance were recorded for the mixture containing 4% nano at the ages of 7, 28, and 90 days, respectively. Farzadnia et al. [136] investigated high-strength mortars containing 0%, 1%, 2%, and 3% of nano-TiO_2_ at different temperatures (28–1000 °C). From the temperature of 300 °C, the decreasing process of compressive strength was observed for the mixtures containing nano-TiO_2_ and the control mixture. They attributed this to the induction of cracks, which is the result of water evaporation. The process of reducing the strength for mixtures containing nanoparticles occurred more gradually than the control mixture, and three reasons were proposed for this process: (1) production of clusters of C-S-H by nano-TiO_2_, (2) amorphous proportion available in nano-TiO_2_, and (3) reduced physically absorbed water due to the presence of nano. In the temperature range of 300 °C to 400 °C, a stable state for compressive strength occurred, which can be related to the transformation of the crystal phase of C-S-H (tobermorite) in this temperature range. From 400 °C to 1000 °C, a sharp decrease in compressive strength occurs, which can be due to the degradation of Portlandite (at 450 °C) and the decomposition of C-S-H (starts at 250 degrees and completes at 750 °C). It was mentioned that up to 300 °C, nano-TiO_2_ reduced the mass loss, but from 300 °C to 1000 °C, no significant difference was seen compared to the control mixture. Guler et al. [137] reported that the use of nano-TiO_2_ in concrete increased its strength. The examination of the control mixture and the mixture containing nano showed that the use of 0.5%, 1%, and 1.5% of nano-TiO_2_ in the amounts of 1.74%, 2.65%, and 3.4% prevents the reduction of residual compressive strength at 300 °C (Figure 23a). Using 0.5%, 1%, and 1.5% of nano-TiO_2_ to the extent of 3.73%, 4.4%, and 4.72% prevented the reduction of residual compressive strength at 500 °C (Figure 23a). Also, at a temperature of 800 °C, the use of 0.5%, 1%, and 1.5% of nano-TiO_2_ prevented the reduction of residual compressive strength by 0.92%, 5.14%, and 7.28% (Figure 23a).

Wang et al. [138] investigated cement mortar 0%, 1%, 2%, 3%, 4%, and 5% of nano-TiO_2_ under low temperatures (0, 5, 10, and 20 °C). It was reported that low temperature leads to a decrease in cement hydration, but the presence of nano-TiO_2_ can improve this defect. By reducing the temperature from 20 °C to 0 °C, the compressive, tensile, and flexural strength decreases at the ages of 3, 7, 28, and 56 days. The best dosage for using nano-TiO_2_ to deal with low temperatures was introduced as 2%. Chunping et al. [137] studied UHPC (containing fly ash and steel fibers) under the effect of using different percentages (0%, 0.5%, 1%, 2%, 3%, 5%) of nano-TiO_2_. They concluded that the presence of nano-TiO_2_ in UHPC leads to a decrease in dry shrinkage, an increase in resistance to chloride ions, and an improvement in resistance to freeze–thaw cycles. They considered a dosage of 1% of nano-TiO_2_ to improve the strength and durability of UHPC. Joshaghani [139] reported that the use of nano-TiO_2_ in concrete led to a decrease in water absorption, a decrease in the height of capillary absorption, a decrease in abrasion mass loss, an increase in electrical resistivity, a decrease in shrinkage, and a decrease in harmful pores. Figure 23b shows the state of the mixture containing 3% nano-TiO_2_ and the control mixture in two fields: shrinkage (90 days) and electrical resistivity (180 days). For more studies on the nano-TiO_2_, see Appendix A.

**Table 3:** A summary of studies based on nano-TiO_2_.

N.O.	n-TiO_2_ (%)	CS	TS	FS	Other Reviews	Results	Refs.
1	0, 3, 5	Y	N	N	Weight loss, Ultrasonic pulse velocity, Radiation transmission, SEM	V-Funnel and L-box: Improving the workability properties (use up to 3%), Improve compressive strength, Improved formation of C-S-H, Reduction of water penetration depth, Reducing the penetration of chloride ions	[121]
2	0, 2, 4, 6, 8	Y	N	N	Impact test, Ultrasonic pulse velocity, Radiation attenuation, SEM	Improving compressive strength (best performance 6% nano), Improved impact strength (best performance 6% nano), Reduction of inner pores, Increasing protective effects against gamma rays	[119]
3	0, 2, 4, 6	Y	N	N	Ultrasonic pulse velocity, Gamma-ray shielding, SEM	With increasing temperature = compressive strength increases first then decreases, With increasing temperature = UPV first increases and then decreases, The better performance of mixtures containing nano compared to the control mixture in terms of the linear attenuation coefficient and compressive strength, Best performance = mixture containing 6% nano	[120]
4	0, 1, 1.5, 3, 5	Y	N	N	X-ray diffraction (XRD), SEM	Improvement of 28-day compressive strength (improvement by 9%), Filling pores, Improvement of the paste-aggregate interface	[122]
5	0, 3, 6, 10	Y	N	N	Modulus of elasticity, MIP test, SEM	Improvement of 28-day compressive strength, A better distribution and refinement of the pores, 10% anatase I mixture and control mixture = highest porosity, Anatase mixture I = the poorest mechanical performance, Best performance = anatase II or rutile, Reduction of concrete rigidity	[123]
6	0, 1, 2, 3	Y	N	N	MIP test, Chloride diffusion	Effective improvement of durability, Increasing the resistance to chloride (the best dosage = 2%).	[124]
7	0, 1	Y	N	N	Chloride diffusion	Increase scouring abrasion resistance, Reducing the chloride ion diffusion coefficient	[125]
8	0.2, 0.4, 0.6, 0.8	N	N	N	Freeze–thaw durability, Nuclear Magnetic Resonance, Industrial CT Scanning, Imaging Test	Preventing pore and crack expansion, Improving freezing and thawing resistance	[126]
9	0, 1, 3, 5	Y	N	Y	Chloride permeability, Pore structure	Improving compressive and flexural strength, Improving and purifying the structure of the pore structure, Improving resistance to chloride penetration	[128]
10	0, 2	Y	N	N	Water absorption, Frost resistance	Improve compressive strength, Decreased water absorption, Improve frost resistance	[129]

CS = Compressive Strength, TS = Tensile Strength, FS = Flexural Strength.

### 7.4. Nano-Silica (Nano-SiO_2_)

Nano silica or silica nano-particles are also known as silicon dioxide particles [140,141,142]. In recent years, the characteristics of nano-SiO_2_ have led to this type of nano being very interesting to researchers. Among the characteristics of nano-SiO_2_, we can mention the following [143,144,145,146,147,148]: (1) high pore volume, (2) optimal surface area, (3) extraordinary potential biocompatibility, (4) harmonic pore size, and (5) the potential to encapsulate hydrophilic/hydrophobic materials. Another thing that can be mentioned is the possibility of managing nano-SiO_2_ in different fields such as shape, particle size, crystallinity, and porosity [143,144]. This wide level of advantages for nano-SiO_2_ has led to it gaining a special place in many different research and industries. For example, the Food and Drug Administration (FDA) has recently approved for the first time a silica-based drug for molecular imaging of cancer [144].

**Figure 24:**
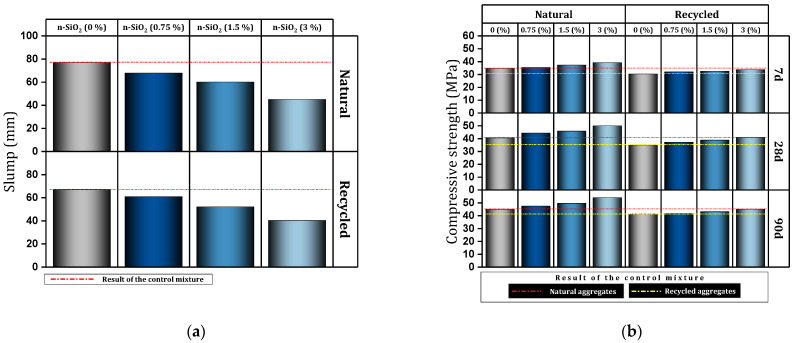
Mixtures containing nano-SiO_2_: (**a**) Effect of nano-SiO_2_ on concrete slump containing natural/recycled aggregate; (**b**) Effect of nano-SiO_2_ on compressive strength of concrete containing natural/recycled aggregate.

Table 4 shows a report of the details and important results of studies conducted on concrete (in some cases cement mortar) containing nano-SiO_2_. Mukharjee and Barai [149] investigated concrete containing different percentages (0%, 0.75%, 1.5%, and 3%) of nano-SiO_2_. They reported that nano-SiO_2_ increased compressive and tensile properties. Also, the significant improvement of non-destructive tests in their study can be mentioned. They also pointed out the role of nano-SiO_2_ in covering the empty spaces of concrete and concluded the improvement of the connection between mortar and aggregate. They also reported a slight improvement in the modulus of elasticity and stated that the reason for this is that the modulus of elasticity is more influenced by the weight of the concrete. In another study, Mukharjee and Barai [150] investigated the effect of nano-SiO_2_ on concrete containing RA. They reported a decrease in a slump with increasing nano-SiO_2_ content and cited the high surface area of colloidal nano-SiO_2_ as the reason (Figure 24a). They reported improved compressive strength at early ages due to pozzolanic activity, as well as improved 28-day compressive strength due to void filling. In the case of the mixture containing 100% RA (without nano-SiO_2_), they reported a 14% decrease in membrane resistance compared to the control mixture, which they attributed to the lower quality of RA compared to virgin aggregates. In this regard, adding 3% nano-SiO_2_ to the mixture containing 100% RA helped to reach a resistance approximately equal to the control mixture (Figure 24b). They pointed out that the ITZ zone in mixes with RA has disappeared to some extent, but the addition of nano-SiO_2_ restores and strengthens this zone and is effective in improving the flexural and tensile strength of these types of concrete.

**Figure 25:**
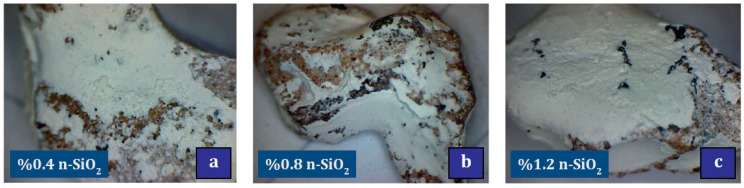
Microscopic images of recycled aggregates under the effect of nano-SiO_2_: (**a**) The mixture contains 0.4% n-SiO_2_; (**b**) The mixture contains 0.8% n-SiO_2_; (**c**) The mixture contains 1.2% n-SiO_2_ [152].

Elrahman et al. [151] studied lightweight concrete containing 0%, 1%, 2%, and 4% nano-SiO_2_. They reported that the use of nano-SiO_2_ (due to its large surface area and small particles) increases the required superplasticizer. Also, the use of doses higher than 1% of nano-SiO_2_ resulted in a significant improvement in the 28-day compressive and flexural strength. The addition of nano-SiO_2_ resulted in a decrease in consistency and an increase in viscosity. Givi et al. [152] studied the effect of different sizes (15nm and 80nm) of nano-SiO_2_ particles on mechanical properties and binary blended concrete. They reported that C-S-H gel formation at early ages is better for nano-SiO_2_ particles with a diameter of 15 nm. This is despite the fact that at older ages, particles with a diameter of 80 nm perform better in this field. They also reported the improvement of compressive, tensile, and flexural strength of concrete due to the use of nano-SiO_2_. Younis and Mustafa [152] investigated the effect of using nano-SiO_2_ in recycled aggregate concrete with an environmental approach. They reported that the compressive strength of the mixture containing 50% RA increased by 10%, 18%, and 22% with the addition of 0.4%, 0.8%, and 1.2% nano-SiO_2_, respectively. Also, the compressive strength of the mixture containing 100% RA increased by 6%, 13%, and 16%, respectively, by adding 0.4%, 0.8%, and 1.2% nano-SiO_2_. Also, a decrease in water absorption of 11% was reported for mixtures containing 0.8%. Among other results of this study, we can mention the help of nano-SiO_2_ in improving the condition of weak and cracked attached mortar and porous ITZ. Figure 25 shows that the presence of nano-SiO_2_ seals the recycled aggregate and fills all surface voids, which can justify the increase in strength of mixtures containing recycled and nano aggregate.

Du et al. [153] in the study of concrete containing nano-SiO_2_ pointed out that the paste morphology at ITZ is more homogeneous in the mixture containing nano-SiO_2_. They also reported that the pore size distribution was modified by nano-SiO_2_, which as a result of this process reduces the ingress rate of water and chloride ions. Also, water penetration depth, chloride migration coefficient, and diffusion coefficient decreased by 45%, 28.7%, and 31%, respectively due to nano-SiO_2_. Kashyap et al. [154] investigated the durability and microstructural of concrete containing nano-SiO_2_ and marble dust. They reported that the use of 2% nano-SiO_2_ along with 5% marble dust leads to a reduction of carbonation depth by about 20%. In general, the acid causes the destruction and deterioration of the calcium hydroxide as well as C-S-H entities, the consequences of which for concrete include mass loss and compressive strength. The combination of nano-SiO_2_ and marble dust has a good effect in controlling the deterioration caused by sulfuric acid. In this regard, the mixture containing 2% nano-SiO_2_ along with 5% marble dust had the least change in weight (6%) and compressive strength (22%) compared to the control mixture.

**Figure 26:**
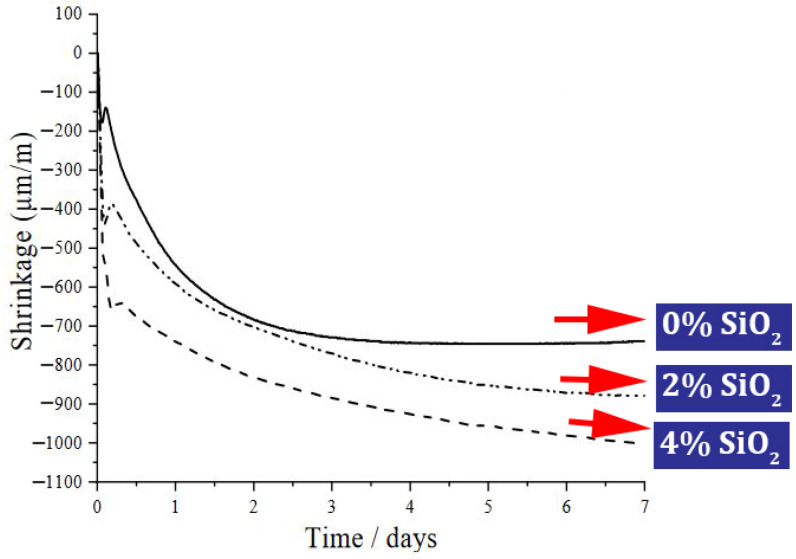
Autogenous shrinkage for mixtures containing 0%, 2%, and 4% nano-SiO_2_ (0–7days) [157].

Moslemi et al. [155] also mentioned the increase in concrete resistance due to the use of nano-SiO_2_ against sulfate attack. They introduced the 8% dose of nano-SiO_2_ as the best choice among the other doses they studied. After 180 days, the mixtures containing 0%, 2%, 4%, 6%, and 8% of nano-SiO_2_ lost 3.51%, 2.4%, 2.23%, 1.13%, and 1% of their weight (relative to the initial weight), respectively. Zhang et al. [156] investigated autogenous shrinkage, hydration heat, and the strength of ultra-high strength concrete (UHSC) containing nano-SiO_2_. They reported improvement in shrinkage with increasing nano-SiO_2_ content. Figure 26 shows the results of autogenous shrinkage of the control mixture and the mixture containing nano-SiO_2_. In this case, it was pointed out that the pozzolanic property of nano-SiO_2_ leads to the reaction with Ca(OH)_2,_ and a denser C-S-H gel is produced, which leads to the reduction of capillary pores and the refinement and modification of the pore structure in concrete. Also, with the increase of nano-SiO_2_ content, the total accumulated heat and compressive strength increased.

**Figure 27:**
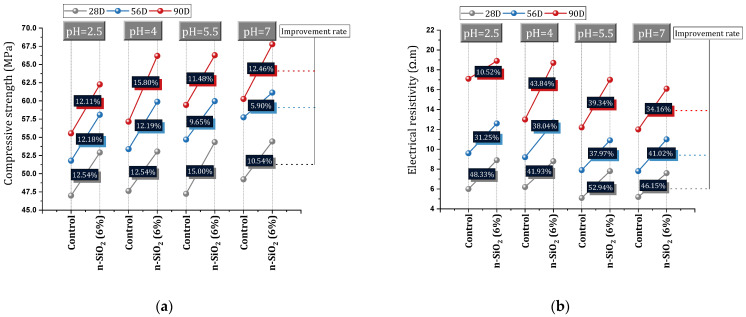
(**a**) Compressive strength of mixtures containing nano-SiO_2_ under different pH. (**b**) The electrical resistivity of mixtures containing nano-SiO_2_ under different pH.

Almohammad-albakkar and Behfarnia [157] studied SCC containing 0, 1%, 2%, and 3% nano-SiO_2_ in the field of drying shrinkage and compressive strength. They reported that nano-SiO_2_ leads to an increase in drying shrinkage in the short term due to the acceleration of the hydration process. In the long term (150 days in this study), due to microstructure modification and C-S-H gel structure optimization by nano-SiO_2_, drying shrinkage decreased. They recommended the use of nano-SiO_2_ up to 2% for field drying shrinkage (5% reduction in drying shrinkage). In this regard, Yu et al. [158] reported that the use of high doses of nano-SiO_2_ can cause a large number of air voids in fresh concrete, and a large number of these voids remain in the hardened state of concrete. As a result of this process, the evaporation of water increases the internal pressure on the capillary network increases, and finally, the concrete will face an increase in drying shrinkage. Mahdikhani et al. [159] investigated the mechanical characteristics and durability of concrete containing 0%, 2%, 4%, and 6% of nano-SiO_2_. To investigate the performance of concrete samples exposed to acid, they cured the samples for 28, 56, and 90 days in an acidic environment with different pH (2.5, 4, 5.5, and 7). They reported that despite the mixes being affected by an acidic environment, the mixes containing nano-SiO_2_ showed higher compressive strength and better water absorption reduction compared to the control mix (Figure 27a). They pointed out that by increasing the content of nano-SiO_2_ due to the high compactness of concrete samples, electrical resistance increases (Figure 27b). As the environment becomes more acidic (i.e., decreasing the pH), the electrical resistance of all samples (with and without nano-SiO_2_) decreases, but the samples containing nano-SiO_2_ show a higher electrical resistance than the control mixture (The best performance of the mixture containing 6% nano-SiO_2_). At the age of 90 days, mixtures containing 6% nano-SiO_2_ obtained 10.52% (pH = 2.5), 43.84% (pH = 4), 39.34% (pH = 5.5), and 34.16% (pH = 7) better electrical resistance compared to the control mixture. At the age of 56 days, the mixtures containing 6% nano-SiO_2_ obtained 31.25% (pH = 2.5), 30.04% (pH = 4), 37.97% (pH = 5.5), and 41.02% (pH = 7) better electrical resistance compared to the control mixture.

**Figure 28:**
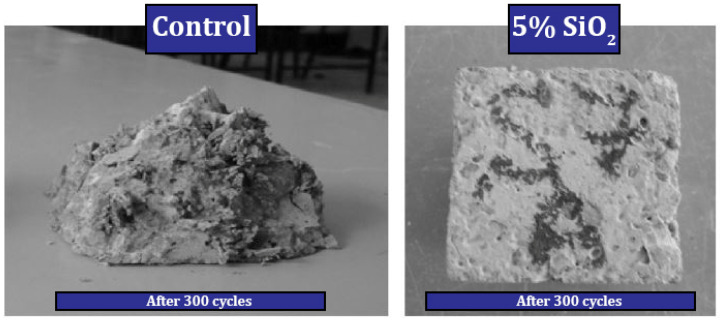
The control sample and the sample containing 5% nano-SiO_2_ after 300 freeze–thaw cycles [161].

Behfarnia and Salemi [160] investigated concrete containing different nanomaterials (nano-SiO_2_ and nano-Al_2_O_3_). Among different percentages (0%, 3%, 5%, and 7%) of nano-SiO_2_ used, they reported the best compressive strength for mixtures containing 5% nano-SiO_2_ (improvement by 14.98%, 30.11%, and 44.98% at the ages of 7, 28, and 120 days, respectively). They also pointed out that the compressive strength of mixtures containing nano-SiO_2_ works better than mixtures containing nano-Al_2_O_3_. Adding nano-SiO_2_ to concrete increased frost resistance. In this regard, they investigated the amount of reduction (compressive strength, change in length, and loss of mass) and increase (water absorption) of different cases in 50, 150, and 300 cycles. They indicated that the mixture containing 5% nano-SiO_2_ gave better performance in the field of frost resistance than other dosages of nano-SiO_2_ for concrete. Figure 28 shows samples without nano-SiO_2_ and samples containing 5% nano-SiO_2_ after 300 freeze–thaw cycles.

**Figure 29:**
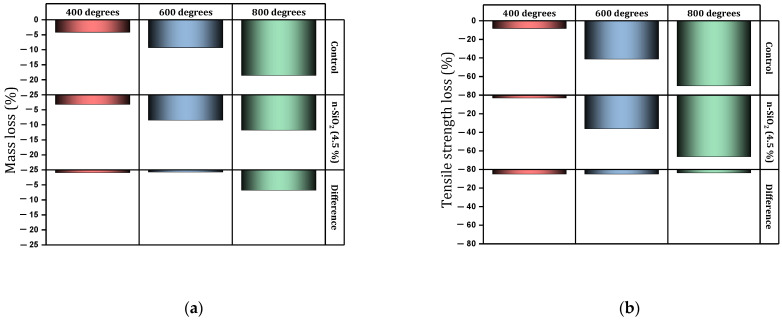
(**a**) The mass loss rate for mixtures containing nano-SiO_2_ under high temperature. (**b**) The tensile strength loss for mixtures containing nano-SiO_2_ under high temperature.

**Figure 30:**
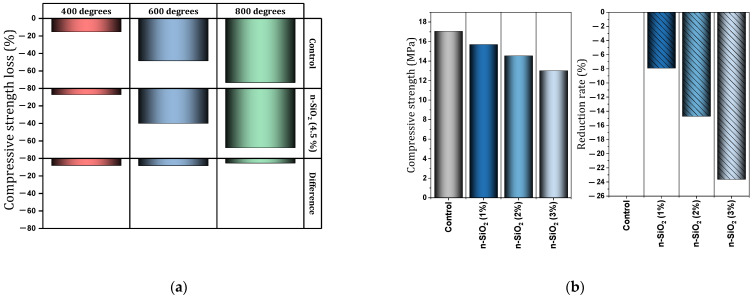
(**a**) The compressive strength loss for mixtures containing nano-SiO_2_ under high temperature. (**b**) Compressive strength of mixtures containing nano-SiO_2_ under the negative temperature (−3 °C).

Tarangini et al. [161] also mentioned the improvement of frost resistance due to the use of nano-SiO_2_. Bastami et al. [162] evaluated HSC containing different percentages of nano-SiO_2_ and silica fume under different temperature conditions (400 °C, 600 °C, and 800 °C). The amount of mass loss for the control mixture (without nano) at 400 °C, 600 °C, and 800 °C was recorded as 4.13%, 9.26%, and 18.56%, respectively, but for the mixture containing nano-SiO_2_ (4.5%), it was 3.20%, 8.84%, and 11.76% (Figure 29a). The amount of decrease in tensile strength for the control mixture (without nano) at the temperature of 400 °C, 600 °C, and 800 °C was 8.14%, 41.19%, and 70.06%, respectively, but for the mixture containing nano-SiO_2_ (4.5%), it was 3.14%, 36.17%, and 66.34% (Figure 29b). Decrease in compressive strength at 400 °C, 600 °C, and 800 °C for the control mixture (without nano) was 15.27%, 48.36%, and 73.28%, respectively, but for the mixture containing nano-SiO_2_ (4.5%), it was 7.14%, 40.01%, and 67.86% (Figure 30a). They indicated that nano-SiO_2_ can effectively improve the mechanical properties of HSC at high temperatures.

Elkady et al. [163] investigated concrete containing 0%, 1.5%, 3%, and 4.5% under elevated temperatures (200 °C, 400 °C, and 600 °C). In the temperature range of 0–400 °C, the compressive strength loss for mixtures containing 0%, 1.5%, 3%, and 4.5% of nano-SiO_2_ was obtained as 30%, 16%, 25%, and 22%, respectively. In the temperature range of 400–600 °C, the compressive strength loss for mixtures containing 0%, 1.5%, 3%, and 4.5% of nano-SiO_2_ was obtained as 49%, 27%, 37%, and 62%, respectively. Also, in the temperature range of 0–400 °C, bond loss for mixtures containing 0%, 1.5%, 3%, and 4.5% of nano-SiO_2_ was obtained as 37%, 29%, 49%, and 26%, respectively. In the temperature range of 400–600 °C, bond loss for mixtures containing 0%, 1.5%, 3%, and 4.5% of nano-SiO_2_ was obtained as 84%, 65%, 79%, and 86%, respectively. They reported that in concrete containing nano-SiO_2_ exposed to high temperature, the deterioration of bond strength occurs faster than the deterioration of compressive strength. Wang et al. [164] examined concrete containing different percentages (0%, 1%, 2%, and 3%) of nano-SiO_2_ under treatment at a negative temperature (−3 °C). Examining the mixtures containing nano-SiO_2_ showed that under the curing conditions of −3 °C, the compressive strength and RCP ability of concrete decreased. For the mixture containing 3% nano-SiO_2_, compressive strength, and RCP ability decreased by 23.63% and 79.84%, respectively. Figure 30b shows the compressive strength results of different mixtures under negative temperature (−3 °C). They stated that under −3 °C processing conditions, nano-SiO_2_ weakens the structure of the ITZ region. In addition, the coarsening degree of pore structure increases with the increase of nano-SiO_2_ content. This is despite the fact that in the conventional curing condition (about 20 °C) based on previous studies, it improves concrete. For more studies on the nano-SiO_2_, see Appendix A.

**Table 4:** A summary of studies based on nano-SiO_2_.

N.O.	n-SiO_2_ (%)	CS	TS	FS	Other Reviews	Results	Refs.
1	0, 0.75, 1.5, 3	Y	Y	Y	Slump, Modulus of elasticity, Ultrasonic pulse velocity, Water absoration, SEM	Slump reduction, Improved compressive and tensile strength, Slight increase in the modulus of elasticity, Significant improvement in non-destructive test results, Improved density, Decreasing water absorption, Reducing the volume of voids	[149]
2	0, 0.75, 1.5, 3	Y	Y	Y	Slump, Ultrasonic Pulse Velocity	Decreasing slump, Improved compressive, tensile, and flexural strength, Improvement of the ITZ region, Significant improvement in non-destructive test results	[150]
3	1, 2, 4	Y	N	Y	Oven-dry density, Thermal conductivity, Specific heat, Water Porosity, Water Absorption, air-void characteristics, MIP test, SEM	Increased superplasticizer required, Significant improvement of resistance and reduction of transport properties, Modification of the air-void system	[151]
4	0, 0.4, 0.8, 1.2	Y	Y	N	Water absorption, SEM	Improving compressive strength: The mixture of 50% recycled aggregate → 0.4% nano (10%↑), 0.8% nano (18%↑), 1.2% nano (22%↑), The mixture of 100% recycled aggregate → 0.4% nano (6%↑), 0.8% nano (13%↑), 1.2% nano (16%↑), Improved tensile strength, Improve water absorption, Improvement of the ITZ region	[165]
5	0, 0.3, 0.9	Y	N	N	Water permeability, Water sorptivity, Water absorption, Chloride migration coefficient, SEM	Improvement of ITZ, Correction of the pore size distribution, Reduction of water penetration depth = 45%, Reduction of chloride migration coefficient = 28.7%, Reduction of diffusion coefficient = 31%	[153]
6	0, 2.5, 5, 7.5, 10	Y	Y	N	Density, Modulus of Elasticity	Increased density, Improved tensile strength Improvement of elasticity modulus and improved compressive strength: 3 days → 3.82–11.84% 7 days → 3.87–17.24% 28 days → 4.93–24.59%	[166]
7	0, 1, 2, 3	Y	N	N	Bulk density, Water absorption, Din permeability, Carbonation, Acid attack, Rapid chloride penetration, SEM, EDS, XRD, TG/DTG	Decreased permeability, Reduce water absorption, Improving resistance in acid attack	[154]
8	0, 2, 4, 6, 8	Y	N	N	Sulfate attack, SEM	Improving compressive strength (especially at early ages), Improving the resistance of concrete against sulfate attack, Improving the microstructure of concrete	[155]
9	0, 2, 4	Y	N	N	Isothermal Calorimetry, Autogenous shrinkage, XRD	Improve compressive strength Improvement of autogenous shrinkage, The increase in the total accumulated heat	[156]
10	0, 1, 2, 3	Y	N	N	Slump, V-funnel, T50, L-box, Drying shrinkage, SEM	Reduction of drying shrinkage, Improve compressive strength, Microstructure improvement	[157]

CS = Compressive Strength, TS = Tensile Strength, FS = Flexural Strength.

### 7.5. The Combined Use of Nanomaterials in Concrete

**Figure 31:**
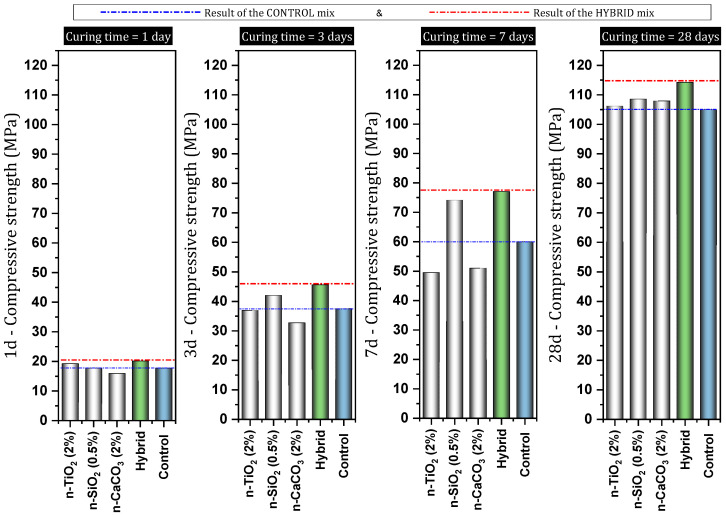
Compressive strength of mixtures containing nanomaterials in hybrid and single.

To benefit from the properties of different nanomaterials at the same time, studies were conducted on the use of two-component and three-component combinations of nanomaterials to make composites. Ren et al. [167] investigated Portland cement paste containing nano-SiO_2_, nano-TiO_2,_ and nano-CaCO_3_. They reported these positive results for a mixture containing all three nanoparticles: (1) more production of hydration products, (2) increased hydration rate, (3) optimized pore structure, and (4) reduced porosity from 23.2% to 16.8%. Also, they reported the improvement of compressive strength for a three-component nano mixture compared to other mixtures. Figure 31 shows the compressive strength results at different ages for the three-component mixture and other mixtures. By the response surface, they predicted the optimal dose of each of the three nanomaterials for the appropriate three-component combination (nano-SiO_2_ in the amount of 0.86 wt%, nano-TiO_2_ in the amount of 2.75 wt% and nano-CaCO_3_ in the amount of 0.14 wt%). Guler et al. [137] investigated concrete mixtures containing nano-SiO_2_, nano-Al_2_O_3_, nano-TiO_2_, and nano-Fe_2_O_3_. They reported that single and hybrid use of nano-SiO_2_ and nano-Al_2_O_3_ has better mechanical properties compared to the other two nanomaterials due to higher pozzolanic activity. The compressive, tensile, and flexural strength were increased by 13.95%, 18.55%, and 21.88%, respectively for the 1.5% mixture of nano-SiO_2_ and nano-Al_2_O_3_. Mohamed [168] also highlighted the notable enhancement in the compressive strength of composite mixtures that incorporate both nano-SiO_2_ and nano-clay, as opposed to mixtures containing only one type of nanoparticle. Shchelokova et al. [169] investigated cement composites containing SiO_2_-TiO_2_ (synthesized by heterocoagulation method). They reported that the temperature of calcination is a very influential factor on the important factors (the particle sizes, crystalline phase, surface area, and photocatalytic activity) of SiO_2_-TiO_2_. It was reported that increasing the temperature of calcination leads to a decrease in the specific surface of the SiO_2_-TiO_2_. The maximum photocatalytic activity was recorded at 800 °C. They introduced the optimal amount of SiO_2_-TiO_2_ (clinicized at 800 degrees) in the range of 0.1–0.5%. By using this optimal value, an increase in compressive strength was obtained at the ages of 1 day (26–29%), 3 days (42–49%), 28 days (41–38%), and 180 days (20–26%). In addition, improvement of abrasion resistance, reduction of water absorption, and reduction of porosity were also reported. Sun et al. [170] reported that the SiO_2_-TiO_2_ mixture offers a better degree of hydration and lower porosity compared to the TiO_2_ mixture.

Sikora et al. [171] evaluated silica-titania nanocomposite. They pointed out that the use of SiO_2_-TiO_2_ structures can ensure the use of the properties of both nanoparticles for the desired mixture. It was reported that in addition to the excellent filling properties that lead to the improvement of the microstructure of the composite, the use of this compound provides optimal performance under UV light (due to the nanomaterial’s self-cleaning and bactericidal properties). They introduced the improvement of the compressive strength of the mixture due to the presence of nano-SiO_2_ and the photocatalytic activity of the mixture due to nano-TiO_2_. Han et al. [172] reported that the use of a SiO_2_-TiO_2_ combination has a good potential to reduce CH crystals in the matrix due to the pozzolanic effect. Also, due to having a self-dispersing effect, they can distribute uniformly in the matrix and achieve a reinforcement/modification effect. Guo et al. [173] reported that the capillary sorptivity (52.22%) and chloride diffusion coefficient (77.43%) were reduced by adding 0.5% of TiO_2_-Graphene to the epoxy resin polymer matrix. 

### 7.6. Nanomaterial Performance in Microstructure

**Figure 32:**
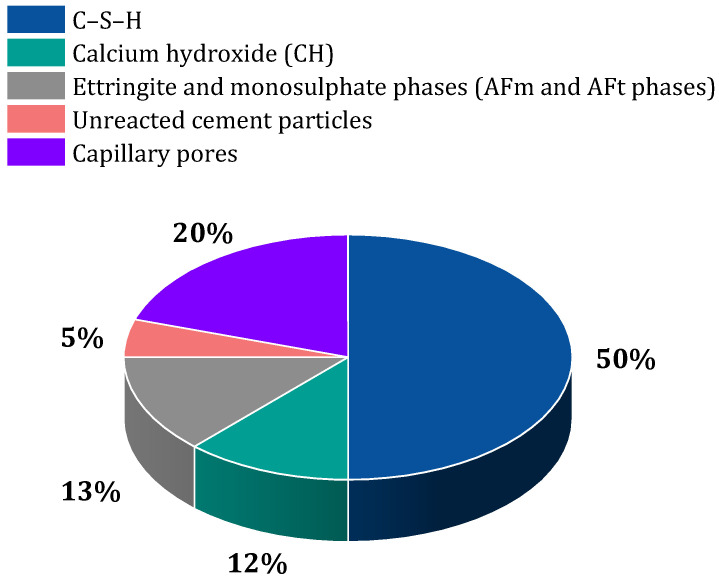
Hydration products in cement concrete.

During the hydration process, a lot of heat is produced, and the main product of this process is calcium-silicate-hydrate (C-S-H) [174]. Concrete structures and elements show slow performance in the field of heat transfer due to low thermal conductivity, which is about 1–1.5 Wm^−1^ K^−1^ [174,175]. This issue becomes more pronounced and sensitive when there is a temperature difference between the surface and the center of the structure, which will cause tensile stress [174]. If the tensile stress exceeds the tensile strength, cracks will appear. These domino-like effects can challenge the durability and more importantly the safety of the structure [176,177,178,179]. The exposure of concrete structures and elements to a temperature higher than 323 Kelvin causes the occurrence of large and disturbing pores in the mortar, which can greatly affect the mechanical, dynamic, and durability characteristics of concrete [180,181]. The chemical and physical characteristics of C-S-H are factors influencing the quality of cement composites and target concrete [182]. Various products are obtained during the concrete hydration process. Figure 32 based on the study of Bensted and Barnes [183] shows a graphical representation of the contribution of each of these products for Portland cement concrete with w/c = 0.5. A major part of hydration products in concrete is C-S-H. Many studies emphasize that the C-S-H gel is one of the important things that can determine the strength and durability of concrete [184,185,186]. The C-S-H gel generally has an amorphous or weakly crystalline structure [42,183,186]. The use of nanomaterials can be one of the effective ways to improve C-S-H defects, which can guarantee better performance against cracking and improve the macroscopic properties of the target concrete [187].

**Figure 33:**
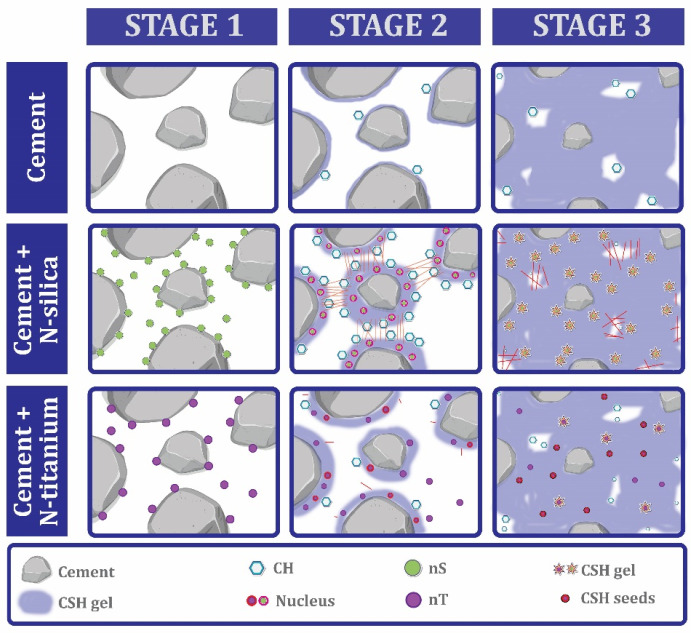
Hydration reaction process.

In silicate chains, electrostatic and bond forces play the main role in developing the strength of the C-S-H structure [188]. In this regard, nanomaterials can improve the mechanism of hydration. Figure 33 shows the hydration reaction process models of mixtures without and containing nanomaterials. In the early ages, C-S-H grains are obtained from the adhesion of nanoparticles to the cement paste, and then C-S-H gel is gradually formed. Nano-SiO_2_ by consuming Ca^2+^ causes the formation of C-S-H seeds [189]. Through the seeding process, the hydration process is followed faster. With the use of C_2_S and C_3_S by nano-SiO_2_, the amount of C-S-H and CH gel increases. CH can also undergo a pozzolanic reaction with nano-SiO_2_, which can reduce CH in the next steps. Nano-SiO_2_ is able to decrease the porosity of C-S-H gel with its function [190]. The reduction of harmful pores by nano-SiO_2_ can be considered as a result of the potential of this type of nano to provide the formation of ettringite with a more network structure between cement particles. The hydration rate of cement is somewhat improved in the presence of nano-TiO_2_ [191]. However, it should be noted that compared to other nanomaterials, it has less power in this field [191]. Nano-TiO_2_ does not have a favorable nucleation effect and this issue can be related to its tendency to consume C_2_S and C_3_S [192]. The presence of nano-TiO_2_ can slow down the precipitation of CH, which causes the structure to increase porosity at the age of 1 day [192]. However, with the passage of time and deposition of nano-TiO_2_ due to the seeding and filling properties, the density of cement pastes increases [189].

**Figure 34:**
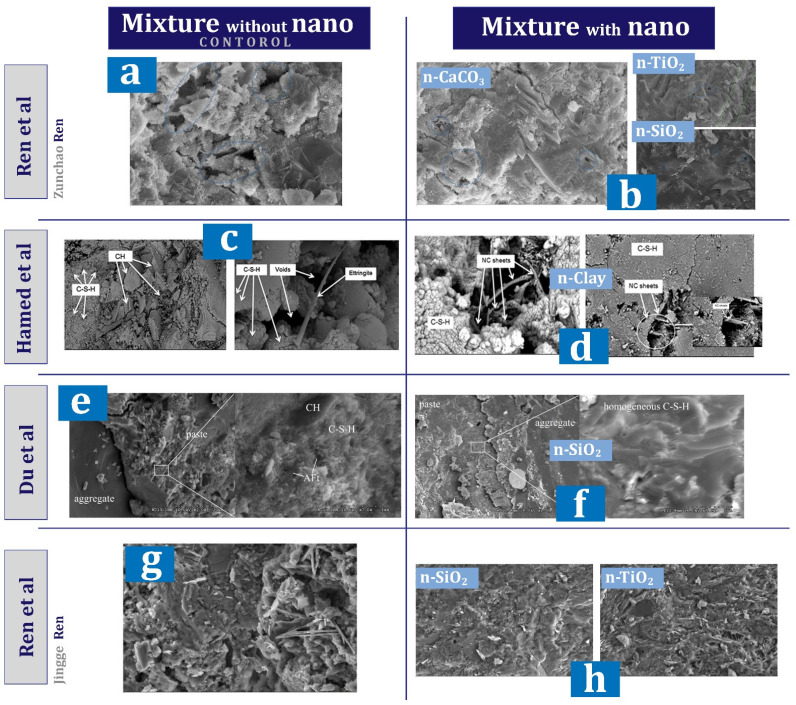
Microstructure analysis of control mixture and mixtures containing nano-CaCO_3_, nano-clay, nano-SiO_2_ and nano-TiO_2_ : (**a**) Control mixture [167], (**b**) Mixtures containing nano-CaCO_3_, nano-TiO_2_, and nano-SiO_2_ [167], (**c**) Control mixture [95], (**d**) Mixtures containing nano-clay [95], (**e**) Control mixture [153], (**f**) Mixture containing nano-SiO_2_ [153], (**g**) Control mixture [122], (**h**) Mixtures containing nano-TiO_2_, and nano-SiO_2_ [122].

Figure 34 shows a report on the microstructure of the control mixture and mixtures containing nano-CaCO_3_, nano-clay, nano-SiO_2_, and nano-TiO_2_ based on the studies of other researchers [95,122,153,167]. Ren et al. [167] pointed out that in the microstructure of the control mixture, the organization of the hydration product is weak and with many holes (more spike-like) (Figure 34a). On the other hand, the microstructure of the mixture containing nano-CaCO_3_, nano-TiO_2_, and nano-SiO_2_ is denser and has much fewer holes (Figure 34b). They attributed this favorable nanomaterial performance to the nucleation effect and pozzolanic properties of these materials. Farokhzad and Divandari [86] also mentioned the improvement of compaction of concrete microstructure and filling of voids by nano-CaCO_3_.

Hamed et al. [95] pointed out that in the mixture without nano-clay, things like the ettringite needles, the calcium hydroxide crystals, and relatively large holes are evident (Figure 34c). It was reported that the calcium hydroxide crystals and the ettringite needles are among the factors that weaken the cement matrix. On the opposite point, nano-clay in the structure of mixtures resulted in benefits such as (Figure 34d) (1) improved consistency and homogeneity and a denser mixture was obtained, (2) significant reduction of space in the matrix, (3) more C-S-H formation due to the reaction of nano-clay with CH residue from the cement hydration process, and (4) preventing cracks by bridging property and having a function similar to fibers.

Du et al. [153] pointed out that in the structure of OPC concrete, things like crystalline CH, needle-shaped ettringite (Aft), and C-S-H along with significant pores were quite evident (Figure 34e). Meanwhile, nano-SiO_2_, due to its pozzolanic activity, by converting CH into secondary C-S-H, has led to the creation of a denser and more homogeneous structure and has minimized the pores (Figure 34f). Rao et al. [193] pointed out that nano-SiO_2_ in concrete mixes has an important effect on the hydration behavior and organization of the produced hydrated products. They noted that in the control mixture, there is a flocculent and sponge-like structure, which is the result of large sheets of Ca(OH)_2_ embedded by the C-S-H gel. The introduction of nano-SiO_2_ in the scenario of micrographs caused the formation of C-S-H hydrates that are completely dense and platelet-like, and on the other hand, it brought continuous and homogenous hydrate crystals. They pointed out that nano-SiO_2_ reacts with CH released during C_3_S hydration to further form C-S-H gel due to its significant pozzolanic activity. It was also pointed out that mixtures containing 3% nano-SiO_2_ show a more dense and homogeneous structure than mixtures containing 4% and 5% nano-SiO_2_. Pourjavad et al. [194] found the main reason for the improvement of flexural strength in their study to be the micro impact and the pozzolanic activity of nano-SiO_2_. Elrahman et al. [151] reported that nano-SiO_2_ led to the densification of concrete microstructure, which they related to the filling effect and the pozzolanic reactivity of this type of nano. They also mentioned that the number of holes has been significantly reduced due to the use of nano-SiO_2_. In this regard, for the control mixture, the total porosity was 54.38 vol.%, but for the mixtures containing 2% and 4% nano-SiO_2_, 51.23 vol.%, and 39.53 vol.% were obtained, respectively.

The reaction of C_4_AF with water leads to the production of C_3_AH_6_. Now, on the other hand, Gypsum (this substance is usually added to it for adjusting the setting time of cement in the production process) reacts with C_3_AH_6_, and ettringite (3CaO·Al_2_O_3_·3CaSO_4_·32H_2_O) is produced [122]. Ren et al. [122] pointed out that because of the ettringite present in the control concrete, needle-like crystals with very large sizes are formed. However, there was no large-size ettringite in the mixtures containing nano-TiO_2_ and nano-SiO_2_, because the nanomaterials used filled the empty spaces to a minimum and refined C-S-H porosities. In mixtures containing nanomaterials, Ettringite cannot grow and damage the structure of the cement paste. According to Figure 34g, the existence of many pores and large crystals is evident for the control mixture. In Figure 34h, the paste-aggregate interface is denser due to the use of nano-SiO_2_ and nano-TiO_2_, which can be a justification for the higher strength of these mixtures compared to the control mixture. Rawat et al. [130] reported that the microstructure examination of the control mixture and the mixture containing 1% nano-TiO_2_ showed relatively large pores (approximately 10 μm). Meanwhile, the mixture containing 2% nano-TiO_2_ shows pores with a size of approximately 2 to 3 μm. Also, mixtures containing 3% nano-TiO_2_ showed relatively fewer pores than the rest of the mixtures. They considered the improvement of the compressive strength of mixtures containing nano-TiO_2_ to be related to the improvement of concrete microstructure. Daniyal et al. [133] reported that the mixture containing nano-TiO_2_ has a better-interlocked structure and less porosity in the ITZ region compared to the control mixture.

The hydration of cement is known as a fibrous character for the calcium silicate hydrate phase (C-S-H) [195,196]. In the structure of cement composites, needle-shaped prismatic crystals are related to the calcium aluminate sulfate hydrate phase (C-A-S-H) [197]. Also, the calcium hydroxide phase (C-H) in the microstructure has crystals with different shapes and sizes [198]. Daniyal et al. [133] pointed out that the mixtures containing nano-TiO_2_ have a small amount of C-A-S-H and C-H but have a large amount of C-S-H. Nano-TiO_2_ increases particle-packing density, significantly reduces porosity, and improves intact bonds for cement composites [199].

### 7.7. Environmental and Economic Performance of Nanomaterials

**Figure 35:**
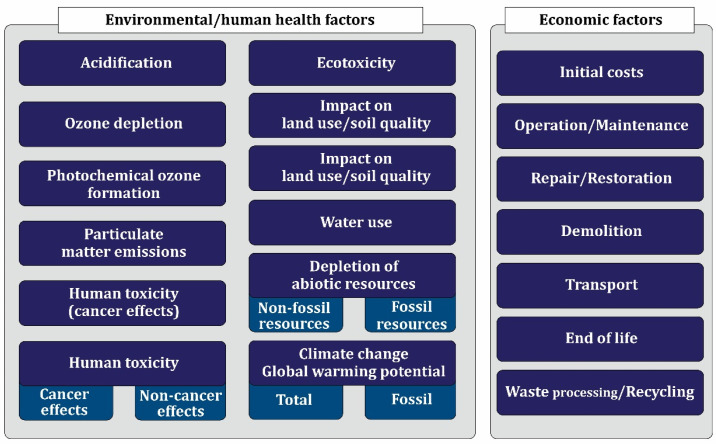
Important factors for analyzing the environmental/economic performance of nanomaterials in concrete based on previous studies.

Examining the technical characteristics of concrete containing nanomaterials (nano-CaCO_3_, nano-clay, nano-TiO_2_, and nano-SiO_2_) indicates that this type of material can be a worthy substitute for cement. Replacing nanomaterials instead of cement can be an effective move in reducing the destructive effects of cement. However, it is important to mention that having good technical characteristics and reducing CO_2_ cannot be a strong justification for the widespread use of this type of material. In this regard, two important items that nanomaterials must pass are (1) environmental performance and (2) economic performance. The proper compatibility of new building materials with the environment should be accompanied by an economic justification, otherwise, there can be little hope for their widespread use. The research performed on the environmental/economic performance of nanomaterials in concrete is very limited. Figure 35 shows a report of the factors that researchers should consider in examining the environmental/economic performance of nanomaterials in concrete [200,201,202,203].

**Figure 36:**
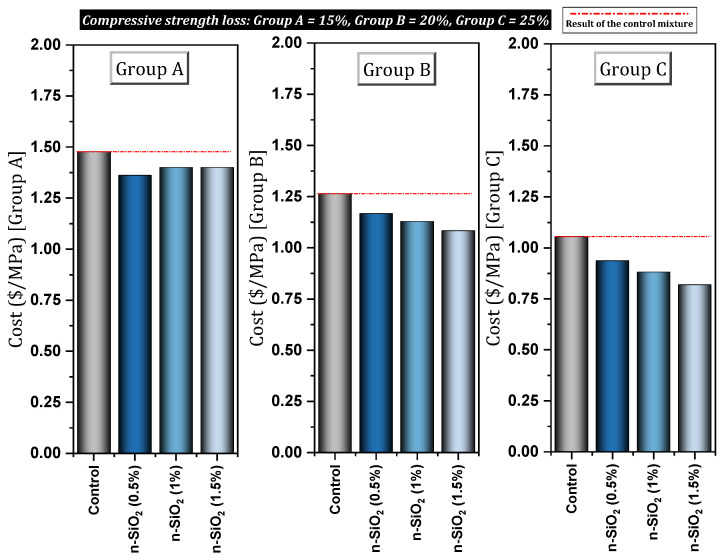
Relationship between concrete compressive strength loss and cost per MPa for different mixes.

**Figure 37:**
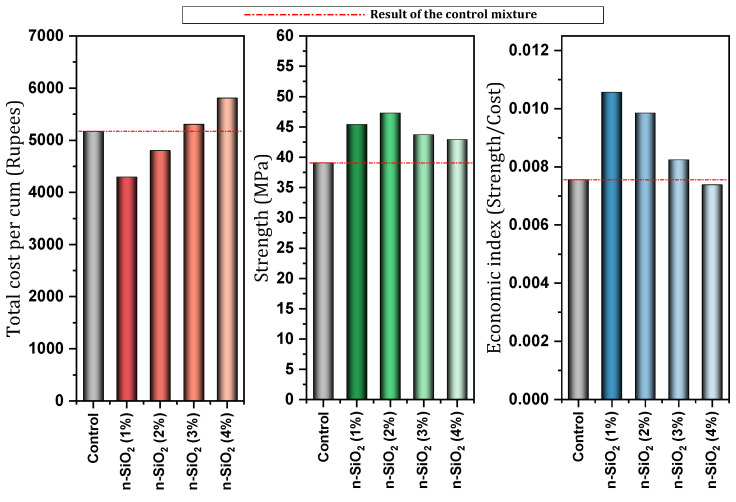
Economic index, strength, and total cost for different mixtures.

Fu et al. [204] investigated the economic analysis of cement composites containing nano-SiO_2_, carbon nanotubes (CNTs), and nanocrystalline cellulose (NCC). They reported that for compressive strength, the lowest cost at 7 d corresponds to mixtures containing nano-SiO_2_ and the lowest cost at 28 d corresponds to CNTs mixtures. For flexural strength, the lowest cost at the age of 7 and 28 days is related to NCC mixtures. Sabour et al. [205] pointed out that the use of 1.5% nano-SiO_2_ in concrete reduced the global warming criterion and fossil fuel consumption by 26.05% and 10.88%, respectively. In addition, the economic evaluation showed that the use of 1.5% nano-SiO_2_ leads to a 10% reduction in life cycle expenses compared to normal concrete. Diab et al. [108] investigated the economic aspect of concrete with and without nano-SiO_2_ exposed to magnesium sulfate attack. In this regard, they performed a simple cost analysis based on the cost of concrete materials ($/MPa) and compressive strength loss at three different levels (15%, 20%, and 25%). Figure 36 shows a schematic view of the results of the economic analysis of the mixtures of this study. They reported that the mixture containing 0.5% nano-SiO_2_ has the lowest cost per MPa in case of a 15% compressive strength loss. Also, the mixture containing 1.5% nano-SiO_2_ has the lowest cost per megapascal in the case of 25% compressive strength loss. Reddy et al. [206] investigated the economic aspect of concrete containing fly ash and nano-SiO_2_. Figure 37 shows the results of different mixtures based on three indicators: total cost, resistance, and economic index. They reported that the mixtures containing 1% and 2% nano-SiO_2_ had more economic value than the control mixture due to their higher strength and lower manufacturing cost. Despite having a better economic value than the control mixture, the mixture containing 3% nano-SiO_2_ does not show good performance due to the higher cost of the mixture than the control mixture. Also, the mixture containing 4% nano-SiO_2_ had higher cost, higher resistance, and lower economic value than the control mixture. Based on cost analysis, they reported the mixture containing 2% nano-SiO_2_ as the best dosage due to its higher strength and high economic index value.

## 8. Bio-Inspired Materials

**Figure 38:**
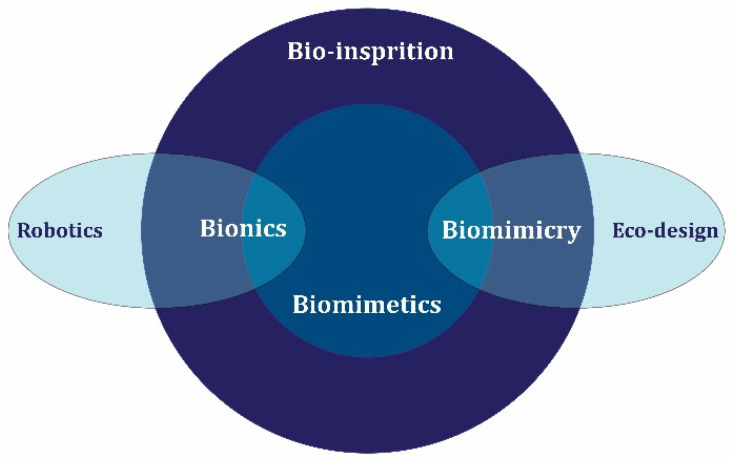
Bio-inspiration and related boundaries.

Nature has always been the teacher of humans. Mankind has achieved important progress and inventions by taking inspiration from nature since long ago. Today, humans also follow the process of getting inspiration from nature in many different industries in a more scientific way and compliance with defined principles. It should be noted that there is a fine line between biomimetic, bionic, and biomimicry [207]. The use of artificial mechanisms to obtain output similar to nature’s function is called biomimetic or biomimicry, but bionic can be considered more related to cybernetics [208,209]. Figure 38 shows a schematic view of Bio-inspiration and linked concepts boundaries map [210].

Researchers have emphasized that biomimicry can improve environmental conditions for buildings [211,212]. Taking inspiration from different aspects (such as structure, behavior, morphology, and performance) of living things in nature can bring effective solutions and new ideas to designers in different building fields [210]. It should be highlighted that for more than 2.1 billion years; multicellular organisms have continued to live and survived the dangers that threatened them [213]. This extraordinary performance of multicellular organisms for survival can promise methods for energy efficiency for structures on Earth or in space.

In general, the construction industry leaves traces of fossil fuel consumption in many of its projects. Increasing sustainability is one of the most important achievements that has gained a more tangible meaning due to the concept of biomimicry in the field of construction processes. But above all, three critical aspects must be considered [214]: (1) technological innovation, (2) environmentally friendly policy, and (3) education. Horn et al. [215] pointed out that based on life cycle assessment (LCA), bio-inspired graded concretes can reduce social burden (37.7%), economic burden (40%), and environmental burden (13%).

The amount of global carbon emissions was recorded as 24.69 GtCO_2_ in 2000 [216]. After the significant reduction of carbon emissions in 2020 due to the important issue of COVID-19, in 2021 the amount of carbon emissions was recorded at 34.9 GtCO_2_, and in 2022 the amount of carbon emissions was recorded at 36.1 GtCO_2_ [217,218,219]. As mentioned in the section “Construction industry in the world”, the construction industry is one of the main factors of pollution of the planet. Chen et al. [220] pointed out that learning about coral reefs’ spatial relationship with their surroundings can bring interesting ideas to engineers. As a real example of comparing the performance of man against the performance of nature, we can refer to the process of making concrete and coral reefs. For its origin, concrete and coral reefs have a common story with CO_2_. In the process of making concrete, components such as aggregate, water, and cement participate. In cement production, mining, transportation, and concrete manufacturing, a large amount of CO_2_ enters the atmosphere. In contrast, the production process of Coral Reef tells a story with a much different ending. In this regard, a high concentration of CO_2_ reacts with water, and carbonate is produced as a result. Calcium in seawater reacts with carbonate and solid calcium carbonate is created for the creation of coral reefs [220,221].

**Figure 39:**
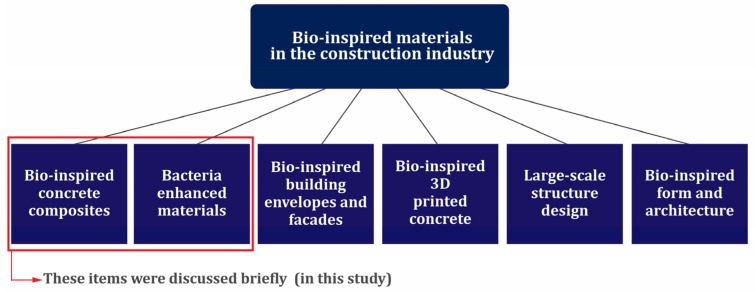
Common fields used for bio-inspired use in the construction industry.

**Figure 40:**
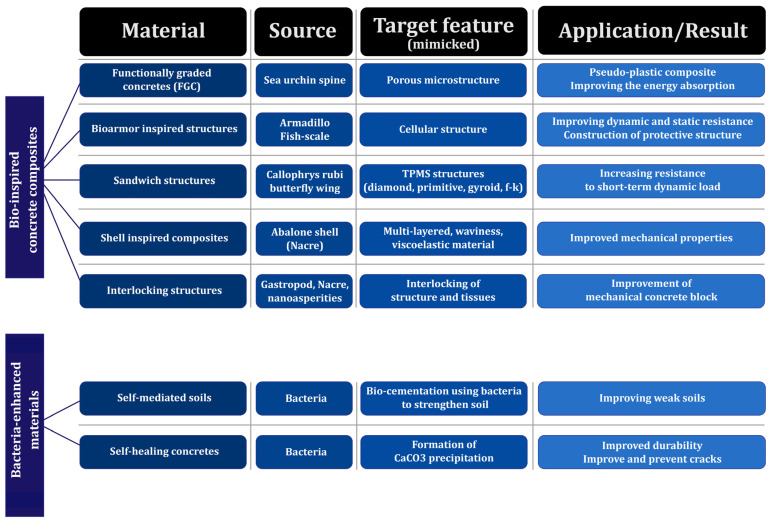
A brief report on bio-inspired materials, composites, and concretes.

The construction industry’s inspiration from nature can be categorized as Figure 39 [222,223]. In this regard, Figure 40 shows a report of composites, concretes, and materials inspired by nature. Figure 40 contains information on Functionally graded concretes (FGC) [224,225], Bio-armor inspired structures [226,227,228,229], Sandwich structures [230,231], Shell inspired composites [232,233], Interlocking structures [234,235], Self-mediated soils [236,237], and Self -healing concretes [238]. Recognition and use of nanostructures in nature have emerged for mankind thanks to progress in the field of nanotechnology. Techniques such as atomic force microscopy (AFM) and scanning tunneling microscopy (STM) have provided the basis for observing nature at the molecular level [239,240]. Making materials with dual characteristics (high adhesion to water and also superhydrophobicity) inspired by the performance of gecko feet is one of the achievements of nanotechnology progress [241,242]. The lotus effect can also be mentioned as another example [243,244]. Today, the use of different nanomaterial synthesis techniques (top-down and bottom-up) has made it possible to synthesize materials inspired by nature in the laboratory [245,246].

The close relationship between nanotechnology and bio-inspiration can lead to very interesting developments and inventions in the future. It seems that man is just at the beginning of a long way and there is still a wide range of things in nature that researchers and scientists have not yet had the opportunity to identify their function. The need to investigate more topologically interlocked structures [247] is felt. Most of the evaluations of laboratory samples are focused on their performance against static load, while the performance of samples against short-term dynamic load is completely different. Therefore, further investigation is necessary to understand the dynamic load behavior of nature-inspired composites and concretes. To do more research in this field, the study of Lazarus et al. [248] can be useful. A major part of the energy emitted from the sun to the earth is wasted by facades or solar panels. Creating a structure to prevent energy reflection and using it more optimally can be very effective. 

## 9. Discussion

The report on the effects of different nanomaterials (nano-CaCO_3_, nano-clay, nano-TiO_2_, and nano-SiO_2_) on 15 concrete characteristics can be found in Table 5, Table 6, Table 7 and Table 8. In this regard, 15 different characteristics include (1) workability, (2) compressive strength, (3) flexural strength, (4) tensile strength, (5) impact strength, (6) water absorption, (7) chloride penetration, (8) carbonation, (9) acid attack, (10) sulfate attack, (11) freeze and thaw, (12) electrical resistivity, (13) elevated temperature, (14) shrinkage, and (15) microstructure. 

Clay has long had a special place in the formation of human societies. Due to its very fine grain structure and features such as pozzolanic reactivity, filling effect, nucleation effect, and needle effect, nano-clay causes purification and refinement of the cement structure. Using nano-clay will reduce workability. In the field of mechanical properties, nano-clay can improve the compressive, tensile, and flexural strength of concrete. The behavior of this type of nano in concrete under dynamic load such as impact load (drop-weight test, projectile impact test, and other cases) is among the cases that need to be investigated and researched. Nano-clay improves the anti-cracking effect and guarantees the reduction of the number of cracks and crack width for concrete. Due to the filling and pozzolanic effect, nano-clay can create good conditions for concrete against acid and sulfate attacks. There is a research gap regarding the effect of nano-clay on carbonation in concrete and cement composites. Also, most of the studies have investigated the shrinkage of cement mortars, and it seems that there is a need to study the effect of nano-clay on concrete shrinkage. At high temperatures, nano-clay can create good conditions for concrete and improve its resistance compared to control concrete. Investigating the behavior of this type of nano in concrete under low temperatures can be further investigated.

Nano-CaCO_3_ provides good conditions for cement structure in terms of reducing capillary porosities and refining pores. This nano creates a favorable improvement for concrete by accelerating the hydration mechanism. The use of nano-CaCO_3_ reduces the workability of mortar. Nano-CaCO_3_, in the field of compressive, tensile, flexural, and impact strength, can also improve the strength of concrete. In the short-term dynamic load field, it seems that there is a need for more studies by researchers. Cement mortars reinforced with nano-CaCO_3_ can experience long-term carbonation and chloride resistance. There is a need for more research in the field of evaluating the use of exclusively nano-CaCO_3_ in concrete under acid and sulfate attacks. The performance of nano-CaCO_3_ in concrete indicates that it can improve the durability of concrete. In this regard, this type of nano provides increased resistance to chloride ion penetration, reduced water absorption, increased resistance to carbonation, and increased resistance to freeze and thaw cycles for concrete. Nano-CaCO_3_ improves the resistance of cement mortar against high temperatures, which is variable in previous studies. In the field of evaluating the performance of concrete containing only nano-CaCO_3_ under high/low temperatures, more research is needed.

Nano-TiO_2_ became more famous for its photocatalytic properties. This nano can increase protective effects against gamma rays. This can improve the quality of concrete exposed to radioactive radiation (such as in hospitals or nuclear power plant structures). Like other nanomaterials, the use of nano-TiO_2_ results in slump reduction. As with many nanomaterials, the use of nano-TiO_2_ results in reduced workability. Refining and improving the microstructure of concrete is also achieved by nano-TiO_2_ (but it does not have a favorable nucleation effect). Under short-term static and dynamic load conditions, nano-TiO_2_ can provide a favorable improvement for concrete (35% improvement). Reducing water absorption, reducing permeability, increasing resistance to freeze and thaw cycles, and resistance to sulfate attack are among the achievements of nano-TiO_2_ for concrete. Also, nano-TiO_2_ increases the resistance of cement mortar against acid attack and carbonation. Evaluation of the resistance of concrete exclusively containing nano-TiO_2_ exposed to acid or carbonation has received less attention and needs further study. At high temperatures, nano-TiO_2_ can act as a contributing factor in improving mechanical properties and preventing mass loss. Investigating the behavior of nano-TiO_2_ at low temperatures is one of the things that can be further investigated.

Nano-SiO_2_ has been used more than any other nanomaterial in the concrete industry. Nano-SiO_2_ usually reduces slump due to the high surface area. Nano-SiO_2_ can homogenize the paste morphology at ITZ and lead to the modification and refinement of the pore size distribution. The use of nano-SiO_2_ in concrete can lead to the provision of favorable concrete against compressive, tensile, flexural, and impact loads. Concrete reinforced with nano-SiO_2_ performs well against non-destructive tests and can guarantee quality concrete. Nano-SiO_2_ leads to the strengthening of high compactness and increases the resistance of concrete against permeability and attacks of harmful ions to a favorable extent. Strengthening frost resistance is one of the other things that nano-SiO_2_ brings to concrete. At high temperatures, nano-SiO_2_ can create better conditions for concrete and prevent resistance loss and mass loss to a favorable extent. At low temperatures, nano-SiO_2_ leads to the weakening of the structure of the ITZ zone and makes concrete suffer from a decrease in strength. More research is suggested on investigating different percentages of nano-SiO_2_ in concrete under low temperatures.

According to the research, depending on which item has more priority for the related project, the dosage of nano is different. However, to achieve the maximum number of items mentioned in Table 5, Table 6, Table 7 and Table 8, the average minimum and maximum dosage according to Table 9 is recommended.

## 10. Conclusions

Nano-CaCO_3_

- Using 1–2% nano-CaCO_3_ can reduce slump by 3.5–14.28%.
- In the early ages of concrete, the presence of nano-CaCO_3_ can significantly increase the compressive strength of concrete. At older ages, there is an improvement in compressive strength, but to a lesser extent than at early ages. Nano-CaCO_3_ can improve tensile strength (19–36%) and flexural strength (17–35%) for concrete.
- The use of 2% nano-CaCO_3_ also improved the behavior of concrete against dynamic load.
- The durability of concrete was improved by using nano-CaCO_3_. Reduction of water absorption (by 17–30%), reduction of chloride penetration (by 20–50%), increased resistance to carbonation (by 66.8%), improvement against acid attacks (reduction of mass loss by 4.2%), improvement against freeze and thaw cycles (at 28 days, 3.6% decrease in compressive strength loss), and improvement in electrical resistance (48.14% at 28 days) are among the things that can be mentioned.
- Using 1% nano-CaCO_3_ can prevent a 76% reduction of mass loss at 800 °C temperature (cement paste).
- The use of nano-CaCO_3_ can effectively reduce capillary porosities and purify pores.

Nano-clay

- The use of nano-clay can lead to a decrease in workability (1–3% nano-clay reduces slump by 3.5–14.5%.)
- Because nano-clay is a rich source of aluminosilicates, it can produce C-A-H and C-S-H gels, which are very effective in improving the mechanical resistance of concrete. The use of 7.5% nano-clay can increase the compressive, tensile, and flexural strength by 24.52%, 9.76%, and 18%, respectively (the use of the Sonicated technique increases the strength by about 1.42–3.47 times).
- Nano-clay can have a positive effect on the durability of concrete. Improving resistance to acid attack (3% nano, prevents 1.6% mass loss), improvement against sulfate attack (9% nano, 41.5% prevention of compressive strength loss), improving resistance to freeze and thaw cycles (improvement by 34%), improving electrical resistance (by 31–38.5%) are among the things that nano-clay brings to concrete.
- When the tempering temperature does not exceed 300 °C, the mixture containing nano-clay shows more resistance compared to the control mixture. The temperature in the range of 440 °C to 450 °C leads to a significant decrease in the compressive strength of concrete containing nano-clay. Reaching a temperature of 800–1000 °C shows a decrease in the compressive strength of mixtures containing clay, but it results in a better situation compared to the control mixture.
- Nano-clay in the structure of concrete provides improvement of the microstructure. Four factors can be mentioned among the improvement features of this type of material: (1) pozzolanic reactivity, (2) filling effect, (3) nucleation effect, and (4) needle effect.

Nano-TiO_2_

- Adding nano-TiO_2_ to concrete can improve protective effects against gamma rays. This capability is very important in nuclear facilities, radioactive waste products transportation, and radiotherapy rooms, which are more exposed to radiation.
- Adding 0.5% of nano-TiO_2_ to the mixture can reduce the slump by 16.34%. Increasing the content of nano-TiO_2_ can result in a decrease in workability.
- The improvement of compressive strength of concrete through nano-TiO_2_ is more obvious at older ages. In the field of tensile and flexural strength, nano-TiO_2_ can improve the strength of concrete. In terms of resistance, nano-TiO_2_ shows a weaker performance compared to nano-SiO_2_. Both nano-TiO_2_ and nano-SiO_2_ CH use cement paste, but it should be noted that the reaction products are different. In this regard, C-S-H produced by nano-SiO_2_ is of high quality and useful.
- The use of nano-TiO_2_ seems to increase the impact resistance of concrete by up to 35%.
- Nano-TiO_2_ can improve the durability properties of concrete acceptably. In this regard, the use of 0.9% of this type of nano improves the resistance against chloride penetration by about 33%. Also, reducing water absorption (1.5% nano, reduction by 6.65%), improving against sulfate attack (3% nano, by 3.87% preventing compressive strength loss), increasing resistance to different freeze and thaw cycles (2% nano improvement in equal to 300 cycles), improvement of electrical resistance (32.86%), improvement of shrinkage (15.23%) was obtained.
- Under high temperatures, nano-TiO_2_ shows acceptable performance. The use of 0.5% to 1.5% of nano-TiO_2_ in a concrete mixture under a high temperature of 800 °C can prevent compressive strength loss by 1% to 7.5%.
- Nano-TiO_2_ covers the pores in C-S-H gel and due to its particle size, it causes adhesive cement to thicken. Nano-TiO_2_ does not have a favorable nucleation effect and this issue can be related to its tendency to consume C_2_S and C_3_S. The presence of nano-TiO_2_ can slow down the precipitation of CH, which causes the concrete structure to increase in porosity at the age of 1 day (this process improves with the passage of time and the deposition of nano-TiO_2_).

Nano-SiO_2_

- Due to the high surface area, nano-SiO_2_ reduces the workability of the fresh mixture (11.55–41.35%).
- At early ages, and more obviously at older ages, nano-SiO_2_ improves the bond between mortar and aggregates and results in an increase in compressive, tensile, and flexural strength.
- Acid causes the destruction and deterioration of the calcium hydroxide and also C-S-H entities. By modifying the pore size distribution, nano-SiO_2_ creates a favorable structure and a good defensive barrier against various ion attacks and permeability. In this regard, 6% nano-SiO_2_ succeeded in increasing the compressive strength of concrete by 12.18% of the compressive strength at pH = 2.5. Also, improving resistance to 300 freeze and thaw cycles, increasing electrical resistance (40%), reducing water absorption (3.21%), reducing chloride ion penetration (29%), and reducing carbonation (23%) are other positive results of nano-SiO_2_.
- Under high temperatures, nano-SiO_2_ shows acceptable performance. The use of 1.5% and 3% of nano-SiO_2_ in concrete mixture under high temperatures (400–600 °C) prevented the reduction of compressive strength by 22% and 12%, respectively.
- Nano-SiO_2_ can homogenize the paste morphology at ITZ and refine the pore size distribution. The pozzolanic property of nano-SiO_2_ leads to a reaction with Ca(OH)_2_ and denser and better C-S-H gel is produced. The formation of C-S-H gel in the early ages is completed by nano-SiO_2_ with smaller particles better than larger particles. In the case of concretes containing recycled aggregates, the ITZ zone is almost destroyed, but the addition of nano-SiO_2_ restores and strengthens this zone.

## 11. Recommendations

Due to the rapid progress of artificial intelligence in the world, the construction industry has also considered the approach of using machine learning (ML) algorithms and deep learning (DL) to provide better-quality concrete/mortar/pate. However, due to the scope of the topics discussed in the field of concrete, it seems that more research is needed. On the other hand, two important cycles that have always contributed to the progress of human society include (1) the cycle of construction and destruction of structures and (2) the cycle of production of agricultural products and production of waste. 

About 48% of the world’s waste is related to construction waste, about 83% of this waste is thrown away and only 17% is recycled. Agricultural production has increased by three times compared to the last 50 years; a large amount of waste is produced in the world every day. On average, about 23.7 million tons of food is produced per day in the world, which usually involves waste.

The use of waste resulting from these two important cycles (construction and agriculture) can be further investigated under the influence of nanomaterials and artificial intelligence algorithms.

## Figures and Tables

**Table 5:** Effect of nano-CaCO_3_ on different parameters of concrete.

N.O.	Characteristic	Recommended Dosages and Effects	Refs.
1	Workability	[1% and 2%] = Slump reduction by 3.5% and 14.28%	[82]
2	Compressive strength	[1%] (early age) = 146–148% ↑ [1%] (90d) = 40% ↑	[82]
		[1%] (300d) ↑	[90]
		[1%, 2%, 3% and 4%] (28d) = 3.75%, 10.73%, 14.47%, and 18.85% ↑	[84]
		[3%] (28d) = 54.9% ↑	[86]
		[3%] (90d) =59.8% ↑	[77]
3	Flexural strength	[1%, 2%, 3% and 4%] (28d) = 17.55%, 24.6%, 26.86%, and 34.63% ↑	[84]
4	Tensile strength	[1%, 2%, 3% and 4%] (28d) = 19.5%, 27.63%, 37.25%, and 35.75% ↑	[84]
		[3%] (28d) = 23.14% ↑	[86]
		[1%] (1–3–7–28d) ↑	[77]
5	Impact strength	[2%] ↑	[85]
6	Water absorption	[1%] (28d) = 17% ↓ [1%] (90d) = 30% ↓	[82]
7	Chloride penetration	[1%] (28d) = 20% ↓ [1%] (90d) = 50% ↓	[82]
		[3%] (300d) = 70.08% improvement in resistance ↑	[90]
8	Carbonation	[3%] (300d) = Normal concrete → 63% improvement in resistance Autoclave concrete → 66.8% improvement in resistance	[90]
9	Acid attack	Examining exclusively nano-CaCO_3_ in concrete = Research gap	---
		[1%] Nano-CaCO_3_ + [20%] Slag = prevented about 4.2% mass loss	[89]
10	Sulfate attack	Examining exclusively nano-CaCO_3_ in concrete = Research gap	---
		[1%] Nano-CaCO_3_ + [45 kg/m^3^] Fly ash = Improve resistance	[88]
11	Freeze and thaw	[5%] (300 cycles) = Improve resistance Reduction of about 83.72% compressive strength loss Reduction of about 26.69% decrease of length Reduction of about 17.17% mass loss Reduction of about 85.99% water absorption	[86]
12	Electrical resistivity	[3%] (28d) = 48.14% ↑ [3%] (90d) = 38.78% ↑	[86]
13	Elevated temperature	Examining exclusively nano-CaCO_3_ in concrete = Research gap	---
		[1%] (800 °C): 76% prevention of mass loss	[87]
14	Shrinkage	[1%, 2%, and 3%] (28d) = 33.95%, 19.45%, and 10.5% ↓	[77]
15	Microstructure	Effectively reduces capillary porosities and refines pores	[82,90]

**Table 6:** Effect of nano-clay on different parameters of concrete.

N.O.	Characteristic	Recommended Dosages and Effects	Refs.
1	Workability	[1%, 2%, 3%, 4% and 5%] = Flow ability reduction by 10%, 13.10%, 16.15%, 19.1%, and 23.52%	[103]
		[1%, 3%, 5%, 7%, 9%, and 10%] = Slump reduction by 6.75%, 8.98%, 10.67%, 12.92%, 14.60%, and 15.73%	[249]
2	Compressive strength	[0.3% and 0.5%] (7–90d) ↑	[91]
		[7.5%] (28d) = 24.52%, 55.47% ↑	[95]
		[1%] (28d) = 35% ↑	[109]
3	Flexural strength	[7.5%] (28d) = 14.97%, 34.97% ↑	[95]
		[1%] (28d) = 31% ↑	[109]
4	Tensile strength	[7.5%] (28d) = 9.76%, 28.05% ↑	[95]
		[1%] (14d) = 34% ↑	[109]
5	Impact strength	Examining exclusively nano-clay in concrete = Research gap	---
6	Water absorption	[1%] (28d) = 54% ↓	[109]
7	Chloride penetration	Examining exclusively nano-clay in concrete = Research gap	---
		Mortar/[1%, 3%, 5%, 7% and 9%] (28d) = Improve resistance: 27%, 29%, 53%, 31%, and 23% ↑	[103]
8	Carbonation	Examining exclusively nano-clay in concrete = Research gap	---
9	Acid attack	Mortar/[3%] = reduction of about 17% compressive strength loss (after 60d)	[104]
		[3%] = Improvement against nitric and sulfuric acid attack (pH = 1) Nitric acid attack (180, 270, and 360d): Reduction of about 0.6%, 1%, and 1.6% mass loss Sulfuric acid attack (60, 120, and 1500d): Reduction of about 2.1%, 2.6%, and 4.1% mass loss	[108]
10	Sulfate attack	[9%] = reduction of about 41.4% compressive strength loss (after 360d)	[108]
11	Freeze and thaw	[3% and 5%] (125 cycles) = good condition, 34% more resistance than control mix	[101,102]
12	Electrical resistivity	[1%] (7d, 28d and 56d) = 31%, 29% and 38.5% ↑	[107]
13	Elevated temperature	[0.3% and 0.5%] (up to 300 °C): Improve resistance and thermal conductivity coefficient	[91]
14	Shrinkage	Examining exclusively nano-clay in concrete = Research gap	---
		Mortar/[1.5% and 3%]: 43% and 40% ↓ Significant reduction (1) number of cracks, (2) crack width, (3) crack length, (4) average cracking area, (5) unit cracking area of each crack	[106,107]
15	Microstructure	Pozzolanic reactivity/filling effect/nucleation effect/needle effect	[95,107]
		Mortar/ increasing C-S-H and decreasing CH	[104]

**Table 7:** Effect of nano-TiO_2_ on different parameters of concrete.

N.O.	Characteristic	Recommended Dosages and Effects	Refs.
1	Workability	[0.5%, 1%, 1.5%, 2%, 2.5% and 3%] = Slump reduction by 16.34%, 21.40%, 31.5%, 42.7%, 50.6%, and 66.6%	[130]
2	Compressive strength	[3%] (28d) = 9% ↑	[122]
		[10%] (28d) = 17.3% ↑	[123]
		[1%] (28d) = 18.04% ↑	[128]
		[2%] (7, 28, 120d) = 12%, 22.71% and 27% ↑	[129]
		[1.5%] (7, 28, 56, 90d) = 18.67%, 6.45%, 10.5% and 7.88% ↑	[130]
3	Flexural strength	[1%] (28d) = 10.28% ↑	[128]
		[1.5%] (7, 28, 56, 90d) = 7.86%, 10.47%, 7.55% and 5.22% ↑	[130]
4	Tensile strength	[1.5%] (7, 28, 56, 90d) = 19.65%, 16.46%, 13.91% and 15.25% ↑	[130]
5	Impact strength	[6%] = 35% ↑	[119]
6	Water absorption	[2%] (28d) = 22% ↓	[129]
		[1.5%, 2%, 2.5%, and 3%] (28d) = 6.45%, 7.45%, 10.3%, and 12.09% ↓	[130]
7	Chloride penetration	[0.9%] = 33% improvement resistance	[124]
		[5%] nano-TiO_2_ + Fly ash = Reduction of 0.639% compressive strength loss Reduction of 0.242% mass loss	[127]
		[1%] (28d) = Improved resistance by 31% ↑	[128]
8	Carbonation	Examining exclusively nano-TiO_2_ in concrete = Research gap	---
		Paste/ [1%, 3% and 5%] nano-TiO_2_ + Fly ash = (28d) reduction of 6.40%, 16.22% and 32.49% carbonation depth ↓ (90d) reduction of 21.26%, 28.18%, and 45.74% carbonation depth ↓ (180d) reduction of 22.92%, 38.39%, and 57.88% carbonation depth ↓	[132]
9	Acid attack	Examining exclusively nano-TiO_2_ in concrete = Research gap	---
		Mortar/ [3% and 5%] = 23.77% and 25.80% improvement in compressive strength (after 360d)	[133]
10	Sulfate attack	[3%] = Reduction of 3.87% compressive strength loss Reduction of 2.381% mass loss	[131]
		[5%] nano-TiO_2_ + Fly ash = reduction of 1.36% compressive strength loss Reduction of 3.293% mass loss	[127]
11	Freeze and thaw	[6%] (25, 50, 75 cycles) = Improve resistance Minimum mass loss (1.52%, 2.66% and 3.99%)	[125]
		[2%] (300 cycles) = Improve resistance Reduction of about 88.5% compressive strength loss Reduction of about 26.4% decrease of Length Reduction of about 78.65% mass loss Reduction of about 7.62% water absorption	[129]
		[1%] nano-TiO_2_ + Fly ash + Steel fiber (0–190 d) = reduction of about 50% mass loss	[250]
12	Electrical resistivity	[3%] (180d) = 32.86 ↑	[139]
		Mortar/ [2%] (3d, 7d and 28d) = 33.70%, 28.50% and 42.50% ↑ [4%] (3d, 7d and 28d) = 93.87%, 77.19%, and 105.61% ↑ [6%] (3d, 7d and 28d) = 183%, 188%, and 189.2% ↑ [8%] (3d, 7d and 28d) = 211.6%, 225.6%, and 313.6% ↑ [10%] (3d, 7d and 28d) = 453.5%, 370%, and 350% ↑	[135]
13	Elevated temperature	Preventing the reduction of residual compressive strength [0.5%, 1%, and 1.5%] (300 °C): 1.74%, 2.65% and 3.4% [0.5%, 1%, and 1.5%] (500 °C): 3.73%, 4.4% and 4.72% [0.5%, 1%, and 1.5%] (800 °C): 0.92%, 5.14% and 7.28%	[137]
		Improve compressive strength [2%, 4%, and 6%] (200 °C): 13.5%, 25.4% and 41.7% [2%, 4%, and 6%] (400 °C): 19.8%, 30.99% and 48.65% [2%, 4%, and 6%] (600 °C): 21.45%, 32.80% and 52.48%	[120]
		Mortar/[1%, 2%, and 3%] nano-TiO_2_ + Silica fume = (28–1000 °C): Improve compressive strength (28–300 °C): Reduce mass loss	[136]
14	Shrinkage	[3% and 5%] (90d) = 15.23% and 43.72% ↓	[139]
		[1%] nano-TiO_2_ + Fly ash + Steel fiber (0–190d) = 9.6% ↓	[250]
		Paste/ [1%, 3% and 5%] + Fly ash (0–28d) = Reduction of 11.03%, 36.03%, and 48.70% ↓	[132]
15	Microstructure	Preventing pore and crack expansion	[126,127]
		C-S-H gel formation, reducing pores, and improving microstructure	[130]

**Table 8:** Effect of nano-SiO_2_ on different parameters of concrete.

N.O.	Characteristic	Recommended Dosages and Effects	Refs.
1	Workability	[0.75%, 1.5% and 3%] = Slump reduction by 11.55%, 12.13%, and 41.35%	[150]
2	Compressive strength	[3%] (7, 28, 90 and 365d) ↑	[149]
		[3%] (7, 28, 90d) = 12–22% ↑	[150]
		[3%] (7 and 28d) = 26% and 31% ↑	[151]
		[10%] (3, 7 and 28d) = 11.84%, 17.24% and 24.59% ↑	[166]
		[3%] (7, 28, 120d) = 8%, 16.67% and 28% ↑ [5%] (7, 28, 120d) = 14.98%, 30.13% and 44.98% ↑	[160]
3	Flexural strength	[3%] (28d) = 14.80% ↑	[150]
		[3%] (7 and 28d) = 18% and 25% ↑	[151]
4	Tensile strength	[3%] (28d) ↑	[149]
		[3%] (28d) = 24% ↑	[150]
5	Impact strength	[10%, 20%, 30%, and 40%] = Improve resistance	[251]
6	Water absorption	[3%] (28d) = 3.21% ↓	[149]
		[3%] = 64.5% ↓	[151]
7	Chloride penetration	[0.9%] (28d) = 29% ↓	[153]
8	Carbonation	[1%] = 23.03% ↓	[252]
		[2%] nano-SiO_2_ + [5%] marble dust = 20% ↓	[154]
9	Acid attack	[2%] nano-SiO_2_ + [5%] Marble dust = minimal change in weight (6%) and compressive strength (22%)	[154]
		Compressive: [6%] (90d) = 12.11% (pH = 2.5), 15.80% (pH = 4), 11.48% (pH = 5.5), and 12.46% (pH = 7) ↑ [6%] (56d) = 12.18% (pH = 2.5), 12.19% (pH = 4), 9.65% (pH = 5.5), and 5.90% (pH = 7) ↑	[159]
		Mass loss: [6%] (90d) = 18.32% (pH = 2.5), 9.40% (pH = 4), 28.97% (pH = 5.5), and 27.55% (pH = 7) ↓ [6%] (56d) = 21.05% (pH = 2.5), 23.76% (pH = 4), 55.1% (pH = 5.5), and 49.27% (pH = 7) ↓	
10	Sulfate attack	[8%] = reduction of about 2.51% mass loss (after 180d)	[155]
11	Freeze and thaw	[5%] (300 cycles) = Improve resistance Reduction of about 83.72% compressive strength loss Reduction of about 26.69% decrease of length Reduction of about 17.17% mass loss Reduction of about 85.99% water absorption	[160]
12	Electrical resistivity	[6%] (90d) = 10.52% (pH = 2), 43.84% (pH = 4), 39.34% (pH = 5.5), and 34.16% (pH = 7) ↑ [6%] (56d) = 31.25% (pH = 2), 30.04% (pH = 4), 37.97% (pH = 5.5), and 41.02% (pH = 7) ↑	[159]
13	Elevated temperature	[1.5%] (0–400 °C): reduction of 14% compressive strength loss [3%] (0–400 °C): reduction of 5% compressive strength loss [1.5%] (400–600 °C): reduction of 22% compressive strength loss [3%] (400–600 °C): reduction of 12% compressive strength loss	[163]
		[4.5%] nano-SiO_2_ + Silica fume = Reduction 8.13% (400 °C), 8.25% (600 °C), and 5.42% (800 °C) compressive strength loss Reduction of 5% (400 °C), 5.02% (600 °C), and 3.72% (800 °C) tensile strength loss Reduction of 0.93% (400 °C), 0.78% (600 °C), and 6.8% (800 °C) mass strength loss	[162]
14	Shrinkage	[2 and 4%] (7d) = 19.33% and 35.37% ↑	[156]
		[2%] (0–150d) = 5% ↓	[157]
15	Microstructure	Improving the condition of weak and cracked attached mortar and porous ITZ	[78,154]

**Table 9:** Minimum and maximum dose of nanomaterials (n-CaCO_3_, n-clay, n-TiO_2_, and n-SiO_2_).

N.O.	Nanomaterials	Min (%)	Max (%)
1	Nano-CaCO_3_	0.5	2
2	Nano-clay	5	7.5
3	Nano-TiO_2_	1	4
4	Nano-SiO_2_	1	5

## Data Availability

The data set analyzed during the current study is available and can be provided upon request.

## Abbreviations

AFM	Atomic Force Microscopy
BC	Before Christ
CAC	Cement Association of Canada
CNT	Carbon nanotube
CS	Compressive Strength
CH crystals	Calcium Hydroxide crystals
CO_2_	Carbon dioxide
CO	Carbon monoxide
Ca^2+^	Calcium ions
C_2_S	Dicalcium silicate
C_3_S	Tricalcium silicate
C_4_AF	Tetracalcium aluminoferrite
C_3_AH_6_	Tricalcium aluminate hexahydrate
C-S-H	Calcium Silicate Hydrate
C-A-S-H	Calcium Aluminate Sulfate Hydrate
CT	Computed Tomography scanning
Ca(OH)_2_	Calcium hydroxide
DNA	Deoxyribonucleic acid
DTA/TG	Differential Thermal Analysis/Thermogravimetry
EDS	Energy Dispersive X-ray Spectroscopy
FS	Flexural Strength
FGC	Functionally Graded Concretes
FTIR	Fourier Transform Infrared Spectroscopy
GtCO_2_	Gigatonnes of carbon dioxide
GHG	Greenhouse gas
UHPC	Ultra-High-Performance Concrete
HPC	High-Performance Concrete
HC	Heavy-Weight Concrete
HSC	High-Strength Concrete
ITZ	Interfacial Transition Zone
LCA	Life Cycle Assessment
MPa	Megapascal
MIP	Mercury Intrusion Porosimetry
NNI	National Nanotechnology Initiative
nm	Nanometer (1 m = 10^9^ nm)
Ni-Cd	Nickel–Cadmium
NS	Nano-SiO_2_
NT	Nano-TiO_2_
NOx	Nitrogen Oxides
NCC	Nanocrystalline cellulose
N	NO (The test was not conducted)
nano-SiO_2_ or n-SiO_2_	nano-sized silicon dioxide
nano-TiO_2_ or n-TiO_2_	nano-sized titanium dioxide
nano-CaCO_3_ or n-CaCO_3_	nano-sized calcium carbonate
nano-Fe_2_O_3_	nano-sized iron oxide
nano-Al_2_O_3_	nano-sized aluminum oxide
OPC	Ordinary Portland Cement
pH	Potential Hydrogen _(a measure of the acidity or alkalinity of a solution)_
QDs	Quantum dots
RCM	Rapid Chloride Migration
RCPT	Rapid Chloride Permeability Test
RA	Recycled Aggregates
SEM	Scanning Electron Microscopy
SCC	Self-Compacting Concrete
SHPB	Split Hopkinson Pressure Bar test
SiO_2_-TiO_2_	A composite material composed of silicon dioxide (SiO_2_) and titanium dioxide (TiO_2_) nanoparticles
T50	T50 test (to measure the flow rate of SCC)
TS	Tensile Strength
UV	Ultraviolet
UHSC	Ultra-High Strength Concrete
USA	The United States of America
UK	The United Kingdom
UPV	Ultrasonic Pulse Velocity
VOCs	Volatile Organic Compounds
W/C	Water-Cement ratio
XRD	X-ray Diffraction
Y	YES (The test was conducted)
°C	Degree Celsius
μm	Micrometer (1 m = 10^6^ µm)
28d, 56d, 90d	28 days, 56 days, 90 days

## Appendix A

**Table A1:** Appendix for Table 1, Table 2, Table 3 and Table 4.

N.O.	Nano	Replacement (%)	CS	TS	FS	Other Reviews	Results	Ref
1	CaCO_3_	0, 1, 2, 3, 4, 5 + (45 kg/m^3^) fly ash	Y	N	N	Modulus of elasticity, SEM, Sulfate attack	Increasing resistance to sulfate attack (best dose = 1%), Improving the microstructure of concrete	[88]
2	CaCO_3_	0, 1, 2 + (20%) Slag	Y	N	Y	Normal consistency, Setting time, Volume stability, Resonance frequency, Thermal conductivity, Acid attack and mass loss, XRD	Improve compressive strength, Improved flexural strength, Resonance frequency improvement, Increasing resistance to acid attack (best dose = 1%)	[89]
3	Clay	0, 1, 3, 6, 9	Y	N	N	Capillary absorption, Total porosity, Expansion strain, Weight loss, UPV, SEM	Reduction of water capillary absorption and total porosity, Improve compressive strength, Improving the resistance of concrete against MgSO_4_ attack, Improving the resistance of concrete against Nitric acid attack and Sulfuric acid attack (at 1.0 pH value)	[108]
4	Clay	0, 1, 2, 3	Y	Y	Y	Water penetration, Water absorption	Improving compressive, tensile, flexural strength (best dose = 1%), Water penetration reduction (best dose = 1%), Water absorption reduction (best dose = 1%)	[109]
5	TiO_2_	0, 1, 2, 3, 4, 5 + (356.12 kg/m^3^) fly ash	Y	Y	Y	Water absorption, Volume of permeable pore space, Sorptivity, Sulfate attack, Chloride attack, SEM, XRD	Improving compressive, tensile, and flexural strength, Reduction of water absorption and volume of permeable pore space, Improving the resistance of concrete exposed to sulfate attack, Improving the resistance of concrete exposed to chloride attack, Improving the microstructure of concrete	[127]
6	TiO_2_	0, 1, 3, 5 + fly ash	Y	N	N	Pore structure, Fluidity, Drying shrinkage, Carbonation, SEM	Carbonation depth reduction, Reduction of drying shrinkage, Improved compressive strength	[132]
7	TiO_2_	0, 2, 4, 6, 8, 10	Y	N	N	Workability, Setting time, Porosity, Water absorption and sorptivity, Electrical resistivity, Rapid chloride permeability, SEM, EDX	Improving the strength of mortar, Improvement of microstructure, Increase in electrical resistance, Porosity reduction	[135]
8	TiO_2_	0, 1, 2, 3 + (5%) silica fume	Y	N	N	UPV, Energy absorption and brittleness, Modulus of elasticity, Mass loss, Gas permeability, SEM, XRD	Improve compressive strength, Positive effect on modulus of elasticity, Reduced permeability, Improvement of mortar structure, Preventing the reduction of mass loss (up to 300 °C)	[136]
9	TiO_2_	0, 0.5, 1, 2, 3, 5 + fly ash + steel fiber	Y	N	Y	Dry shrinkage, Chloride diffusion, Freeze–thaw, Carbonation, MIP	Improving the strength of concrete, Reduction of dry shrinkage, Increased resistance to chloride ion, Improved resistance to freeze–thaw cycles	[250]
10	TiO_2_	0, 3, 5	Y	Y	Y	Slump, Water absorption, Freeze and thaw, Abrasion resistance, Bulk electrical resistivity, Modulus of elasticity, Pore size distribution, MIP	Improving the strength of concrete, Reduce water absorption, Reducing the height of capillary absorption, Reduce abrasion weight loss, Increase in electrical resistivity, Reduction of shrinkage, Reduction of harmful pores	[139]
11	TiO_2_	0.4, 0.8, 1.2	N	N	N	Capillary-water-absorption test after freeze–thaw cycles, Pore Test, SEM	Reduce the cumulative water absorption and porosity of recycled aggregate, Filling the gap between the aggregate, Enhancing the frost resistance, Enhance the interfacial bonding force between aggregate and cement	[253]
12	TiO_2_	0, 0.5, 1, 1.5, 2, 2.5, 3	Y	Y	Y	Slump, Sorptivity, Water absorption, UPV, Dynamic modulus of elasticity, Chloride penetration, SEM	Improving compressive, tensile, and flexural strength, Reducing water absorption and apparent porosity, Reduction of chloride penetration, Improvement of concrete microstructure	[130]
13	TiO_2_	0, 3	Y	N	N	Ultrasonic Wave Velocity, Mass loss, Sulfate attack, XRD	Increasing the resistance of concrete against sulfate attack, Reduce mass loss, Reduce compressive loss, Improving the microstructure of concrete	[131]
14	TiO_2_	0, 1, 3, 5	Y	N	N	Setting time, Potentiodynamic polarization, SEM, XRD, EDS	Improve compressive strength, Improving the resistance of composite exposed to the acidic environment, Reducing the corrosion rate, Reduction of pores and production of C-S-H gel, Increasing particle-packing density	[133]
15	TiO_2_	0, 0.5, 1, 1.5	Y	Y	Y	SEM, XRD	Improving compressive, tensile, and flexural strength, Preventing the reduction of residual compressive strength at high temperatures, Improvement of concrete microstructure	[137]
16	SiO_2_	0, 2, 4, 6	Y	N	N	Weight loss, Electrical resistance, Water absorption	Obtaining a higher compressive strength than the control mixture (even under the influence of acid), Better performance in reducing water absorption than the control mixture (even under the influence of acid), Increase in electrical resistivity by increasing nano-SiO_2_ content	[159]
17	SiO_2_	0, 3, 5, 7	Y	N	N	Water absorption, Frost resistance (freeze and thaw cycle)	Improve compressive strength, Decreased water absorption, Improving frost resistance (in all 50, 150, and 300 cycles)	[160]
18	SiO_2_	0, 1.5, 3, 4.5 + (0–22.5 kg/m^3^) Silica fume	Y	Y	N	Compressive strength, Tensile strength, Mass loss	Improved tensile strength (at high temperature), Improved compressive strength (at high temperature), Preventing weight loss of concrete at high temperatures, Better performance of nano-SiO_2_ than silica fume	[162]
19	SiO_2_	0, 1, 2, 3	Y	N	N	Resistance to chloride ion penetration, Nuclear magnetic resonance, SEM, XRD	Under curing conditions −3 °C: Reducing compressive strength, Decreased RCP ability, Weakening of ITZ, and increasing the coarsening degree of pore structure	[164]
20	SiO_2_	0, 1.5, 3, 4.5	Y	N	N	Pull out, Water permeability, SEM, XRD	Improved compressive strength (at high temperature), Improved bond strength (at high temperatures), Improving the microstructure of concrete (at high temperatures)	[163]
